# DELTA: a method for brain-wide measurement of synaptic protein turnover reveals localized plasticity during learning

**DOI:** 10.1038/s41593-025-01923-4

**Published:** 2025-03-31

**Authors:** Boaz Mohar, Gabriela Michel, Yi-Zhi Wang, Veronica Hernandez, Jonathan B. Grimm, Jin-Yong Park, Ronak Patel, Morgan Clarke, Timothy A. Brown, Cornelius Bergmann, Kamil K. Gebis, Anika P. Wilen, Bian Liu, Richard Johnson, Austin Graves, Tatjana Tchumatchenko, Jeffrey N. Savas, Eugenio F. Fornasiero, Richard L. Huganir, Paul W. Tillberg, Luke D. Lavis, Karel Svoboda, Nelson Spruston

**Affiliations:** 1 Janelia Research Campus, Howard Hughes Medical Institute, Ashburn, VA USA; 2 Department of Neurology, Feinberg School of Medicine, Northwestern University, Chicago, IL USA; 3 Institute for Experimental Epileptology and Cognition Research, Medical Faculty, University of Bonn, Bonn, Germany; 4 Solomon H. Snyder Department of Neuroscience, Johns Hopkins University School of Medicine, Baltimore, MD USA; 5 Department Institute of Neuro- and Sensory Physiology, University Medical Center Göttingen (UMG), Göttingen, Germany; 6 Department of Life Sciences, University of Trieste, Trieste, Italy; 7 Kavli Neuroscience Discovery Institute, Johns Hopkins University School of Medicine, Baltimore, MD USA; 8 Allen Institute for Neural Dynamics, Seattle, WA USA

**Keywords:** Learning and memory, Synaptic plasticity

## Abstract

Synaptic plasticity alters neuronal connections in response to experience, which is thought to underlie learning and memory. However, the loci of learning-related synaptic plasticity, and the degree to which plasticity is localized or distributed, remain largely unknown. Here we describe a new method, DELTA, for mapping brain-wide changes in synaptic protein turnover with single-synapse resolution, based on Janelia Fluor dyes and HaloTag knock-in mice. During associative learning, the turnover of the ionotropic glutamate receptor subunit GluA2, an indicator of synaptic plasticity, was enhanced in several brain regions, most markedly hippocampal area CA1. More broadly distributed increases in the turnover of synaptic proteins were observed in response to environmental enrichment. In CA1, GluA2 stability was regulated in an input-specific manner, with more turnover in layers containing input from CA3 compared to entorhinal cortex. DELTA will facilitate exploration of the molecular and circuit basis of learning and memory and other forms of plasticity at scales ranging from single synapses to the entire brain.

## Abstract (web summary)

Researchers developed DELTA, a method for brain-wide measurement of synaptic protein turnover with single-synapse resolution, providing a powerful tool to localize and study mechanisms underlying synaptic plasticity and learning.

## Main

Cellular functions are tuned by protein synthesis and degradation, which results in dynamic protein turnover^1–3^. In the brain, protein lifetimes range from tens of minutes for immediate-early gene proteins^4^ to months^5–7^. Synthesis and degradation rates of proteins vary by protein, cell type and brain region^8–10^ and can be modulated by neuronal activity^11^. Protein dynamics also depend on environmental and behavioral conditions^10,12^ that are necessary for animals to learn cognitive and motor tasks^13–15^, and are dysregulated in neurodegenerative diseases^16^. Learning is supported by long-term changes in synaptic strength that require both protein synthesis^17,18^ and degradation^19–21^. Since behavior-related plasticity occurs in a coordinated manner across multiple brain regions^22^ but may be limited to specific synapses in individual neurons^23–25^, measurements of protein turnover are needed at multiple spatial scales, ranging from brain wide to subcellular.

Metabolic incorporation of stable-isotope-labeled amino acids followed by mass spectrometry (MS) allows the measurement of turnover for many proteins in parallel^26^, but its spatial resolution is limited to the level of brain regions (for example, cortex versus cerebellum) or homogenate fractions (for example, cytoplasm versus synaptosomes). Recently, a suite of new tools^27–31^ has enabled proteins of interest to be labeled with fluorescent markers and imaged with high contrast and resolution, including a pulse-only^29^ method using the HaloTag^32^ (HT) and fluorescent ligands (Supplementary Text).

Here, we identified a panel of HaloTag ligand (HTL) Janelia Fluor (JF) dyes^33–36^ that are optimized for brain-wide pulse–chase labeling of synaptic proteins in HT knock-in mice. We used these JF-HTL dyes to develop DELTA (Dye Estimation of the Lifetime of proTeins in the brAin), a method that measures protein dynamics in vivo using pulse–chase experiments with spectrally separable fluorescent ligands. DELTA enables precise measurements of protein lifetime in the whole brain and other tissues in vivo, down to synaptic resolution. We applied DELTA to three knock-in mouse lines expressing the HT protein, including a newly generated glutamate receptor subunit (GluA2) HT knock-in mouse, a widely expressed AMPA-type glutamate receptor subunit^37,38^, which allowed us to map learning-related synaptic plasticity on a brain-wide scale. In a visually guided foraging task, GluA2 turnover was elevated in hippocampal CA1, with higher GluA2 turnover in dendrites receiving input from hippocampal CA3 compared to entorhinal cortex. A more widespread pattern of synaptic protein turnover was seen in response to environmental enrichment.

## Results

### Modeling and measuring protein turnover in vivo

**Fig. 1:**
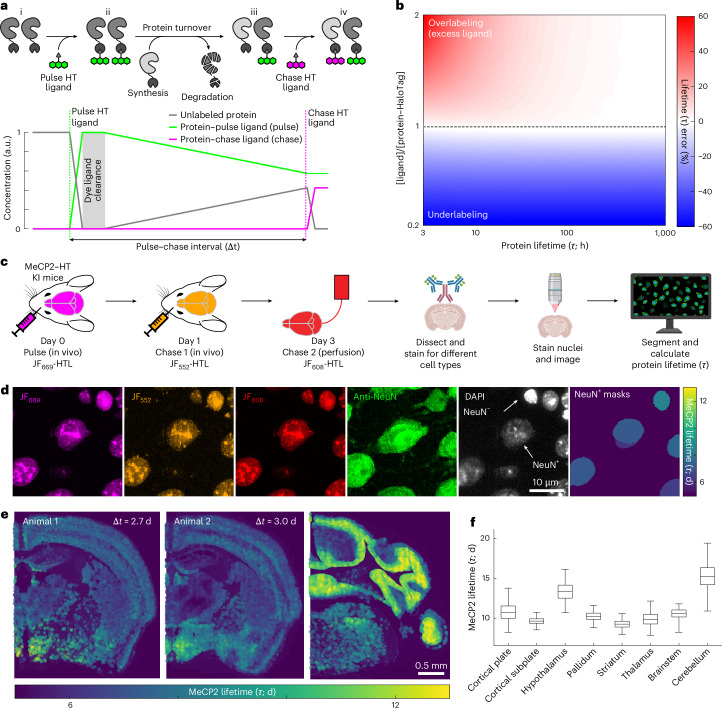
Measurement of protein turnover in vivo*.* **a**, DELTA measures protein lifetime by sequential HTL dye capture using a HT-modified protein. (i) Before the injection of the first dye ligand (pulse), all proteins are unlabeled (gray line in graph). (ii) After injection of the pulse dye ligand (dashed green line), all proteins are labeled with the pulse dye ligand (solid green line, pulse). (iii) During the pulse–chase interval, some proteins degrade, and others are synthesized but are unlabeled. (iv) Injection of a spectrally separate chase dye ligand (dashed magenta line) binds the newly synthesized protein (solid magenta line, chase). The gray shaded area indicates where excess dye ligand delays the onset of turnover measurement, leading to a pulse overestimation error (Supplementary Text). **b**, The estimated lifetime error (color) as a function of dye-protein ratio (*y* axis) and true protein lifetime (*x* axis). Undersaturation (<1 dye ligand–protein ratio) causes worse errors than dye ligand excess and longer-lived proteins are estimated more accurately than short-lived ones. **c**,**d**, Turnover measurement of the nuclear protein MeCP2–HT in a knock-in (KI) mouse model. **c**, Experimental design: Three HTL dyes were used to measure multiple protein turnover intervals. After perfusion and dissection, coronal sections were labeled with DAPI and fluorescent antibodies to identify different cell types. **d**, Example field-of-view images showing the JF dyes with NeuN and DAPI for identification of neuronal nuclei. After segmentation of NeuN-positive nuclei, segmented nuclei were colored by lifetime using the sum of the two in vivo injections as the pulse (fraction pulse = (JF_669_ + JF_552_)/(JF_669_ + JF_552_ + JF_608_)). **e**, Example coronal sections from two animals. Left and middle: images show the consistency of the lifetime estimates for aligned anteroposterior sections. Right: image shows the longer lifetime in the cerebellum (compared to middle and left images). **f**, MeCP2–HT neuronal nuclei lifetime (bootstrap of means from five animals and three intervals where the line is the median, boxes denote the 25th–75th percentiles and whiskers mark the 0.5th–99.5th percentiles) across CCF-aligned brain regions. a.u., arbitrary units.

**Extended Data Fig. 1:**
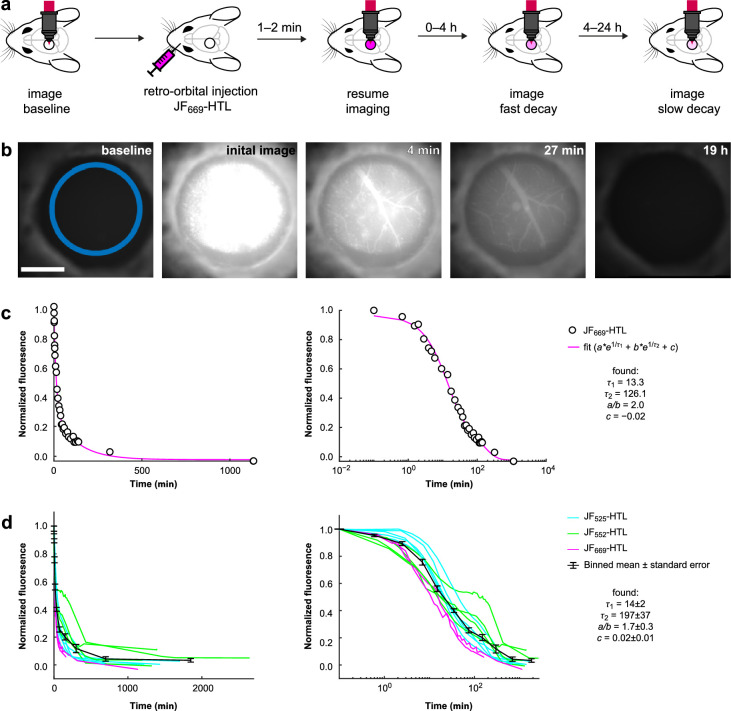
HaloTag ligand dyes are cleared from the brain within hours. **a**, Experimental procedure to measure dye-ligand clearance. The rise in fluorescence is not captured, as the animal is not imaged during the injection. **b**, Example images for a JF_669_-HTL injection. The blue circle represents the region for which fluorescence was quantified in (c). Scale, 2 mm. **c**, Linear (left) and log (right) fluorescence (peak normalized and baseline subtracted) as JF_669_-HTL is cleared from the brain. Double exponential fit in red; *τ*_1_ and *τ*_2_ are in minutes. **d**, Similar data for 12 experiments with **3** dyes (JF_525_-HTL in green, JF_552_-HTL in red, and JF_669_-HTL in magenta). The black trace is a binned average, with standard error for all **12** experiments. The numbers in (d) represent the mean ± standard error of double exponential fits for the 12 experiments.

We first modeled the HT dye ligand labeling process to identify conditions under which a pulse–chase method would enable precise protein turnover measurements in vivo of a HT-fused protein (Fig. 1a). We considered three populations of HT fusion proteins: (1) unlabeled protein–HT; (2) pulse, the population labeled with the first dye ligand infusion; and (3) chase, the newly synthesized protein–HT population labeled with a second infusion using a spectrally distinct dye ligand. Assuming an exponential decay of these protein populations and stable protein concentrations, we can calculate their mean lifetime *τ* = Δ*t*/log(1/fraction pulse), where Δ*t* is the time interval between pulse and chase dye ligand administration and fraction pulse is the proportion of protein–HT fusion labeled by the pulse dye ligand (fraction pulse = pulse / (pulse + chase)). As slow dye ligand clearance affects turnover measurements (Fig. 1a and Supplementary Text), we measured dye ligand clearing using in vivo imaging and determined an effective time constant of approximately 82 min (Extended Data Fig. 1).

**Extended Data Fig. 2:**
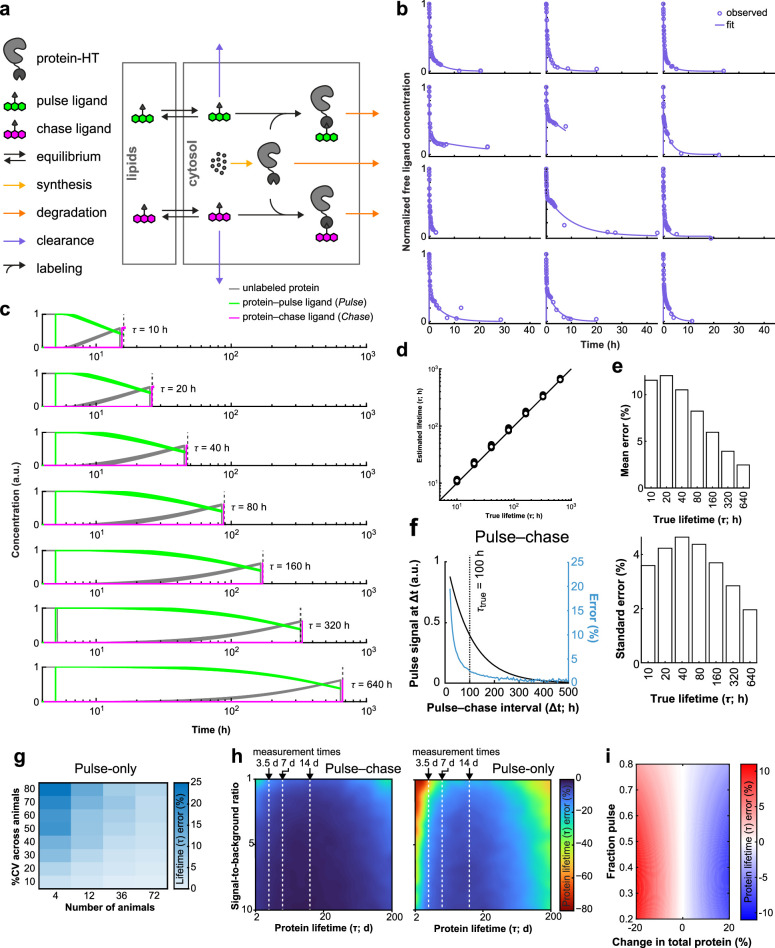
Modeling pulse-chase experiments *in vivo*, constrained by dye-ligand clearance measurements. **a**, Model compartments, molecules, and reactions for modeling protein turnover measurements. A pulse or chase dye-ligand is added to the cytosol compartment and subsequently: (1) cleared; (2) can move to a lipid compartment (representing the slow dye-ligand clearance component); or (3) irreversibly binds to the protein-HT. Here, the protein synthesis rate is equal to its degradation rate, with dye-bound proteins degrading at the same rate. This maintains total protein amount constant regardless of dye-ligand binding. See Supplementary Text for more information. **b**, Fitting of the dye-ligand clearance data (Extended Data Fig. 1) to generate model variants. Twelve experiments (data in blue circles) and the fitted response (blue lines) are shown. The parameters fit were dye-ligand clearance rate, cytosol-to-lipid rate constant, and volume of the lipid compartment. The cytosol compartment was fixed to having a volume of 1 ml. **c-e**, Example model simulations with a selection of 7 HT-protein mean lifetimes (c: 10-640 h; Legend as in Fig. 1a). The correlation between estimated and true protein lifetime is excellent (d). The mean error (e, top) decreases with increasing lifetime, whereas the standard error between model variants (simulating variability in dye-ligand clearance) peaks around the mean dye-ligand clearance rate (e, bottom). **f**, Simulation of error in the lifetime estimate as a function of the pulse-chase interval (∆T). The error (blue line) decreases faster than the *Pulse* concentration (black line). This shows that the error with DELTA is dominated by dye-ligand clearance, with overall low error rates. **g**, Error in lifetime estimation using pulse-only measurements, given ideal dye-ligand injection delivery. Here, variability of the measurement across animals (CV y-axis) could be countered only by averaging across animals (number of animals x-axis). Error rates (color-coded) are higher than in DELTA, where the chase dye-ligand and the *Fraction Pulse* calculation normalizes for inter-animal variability under ideal dye-ligand injection conditions. **h**, Modeling to compare errors due to shot noise with DELTA (left panel) *vs*. pulse-only labeling (right panel). In both cases three measurement times (white dashed lines) were used to estimate a known decay (x-axis: 2-200 days). Increasing the signal to background ratio (y- axis) and adding a chase dye-ligand (as in DELTA; left panel) reduced shot-noise induced errors. **i**, Modeling the effects of a change in total protein on the error in lifetime estimation (% error in colormap) as a function of % change in protein levels (x-axis) and the measured fraction pulse (y-axis). Error increases with longer intervals (smaller fraction pulse) and larger changes in protein.

Using modeling and in vivo dye ligand clearance measurements, we investigated the effects of the three experimentally controlled variables on estimates of lifetime using DELTA: (1) the selection of a target protein, which determines mean lifetime; (2) the amount of pulse dye ligand injected, which determines ligand-to-target ratio; and (3) the pulse–chase interval (Δ*t*; Fig. 1b, Extended Data Fig. 2 and Supplementary Text). We found that under-labeling (less dye ligand than target protein) produced large errors, regardless of lifetime. However, dye ligand excess produced only small errors over a large range of lifetime values, except for proteins where lifetime is on the order of or shorter than dye ligand clearance (Fig. 1b). As most proteins in the brain have lifetimes far longer than dye ligand clearance times (lifetimes from days to weeks; effective clearance time constant of ~82 min)^1,10^, dye ligand excess is the preferred regime. Additionally, dye ligand excess would not distort relative measurements of protein lifetime across cell types, brain regions or individual animals. Compared to single-dye methods (pulse-only)^29^, the normalization provided by the chase dye ligand in DELTA increased the signal-to-noise ratio to measure turnover (Extended Data Fig. 2g,h and Supplementary Text). Protein levels can be tracked by summing the pulse and chase. In cases where protein levels change, that is, synthesis and degradation are out of balance, corrections need to be applied to the protein lifetime estimation outlined above (Methods and Extended Data Fig. 2i).

**Extended Data Fig. 3:**
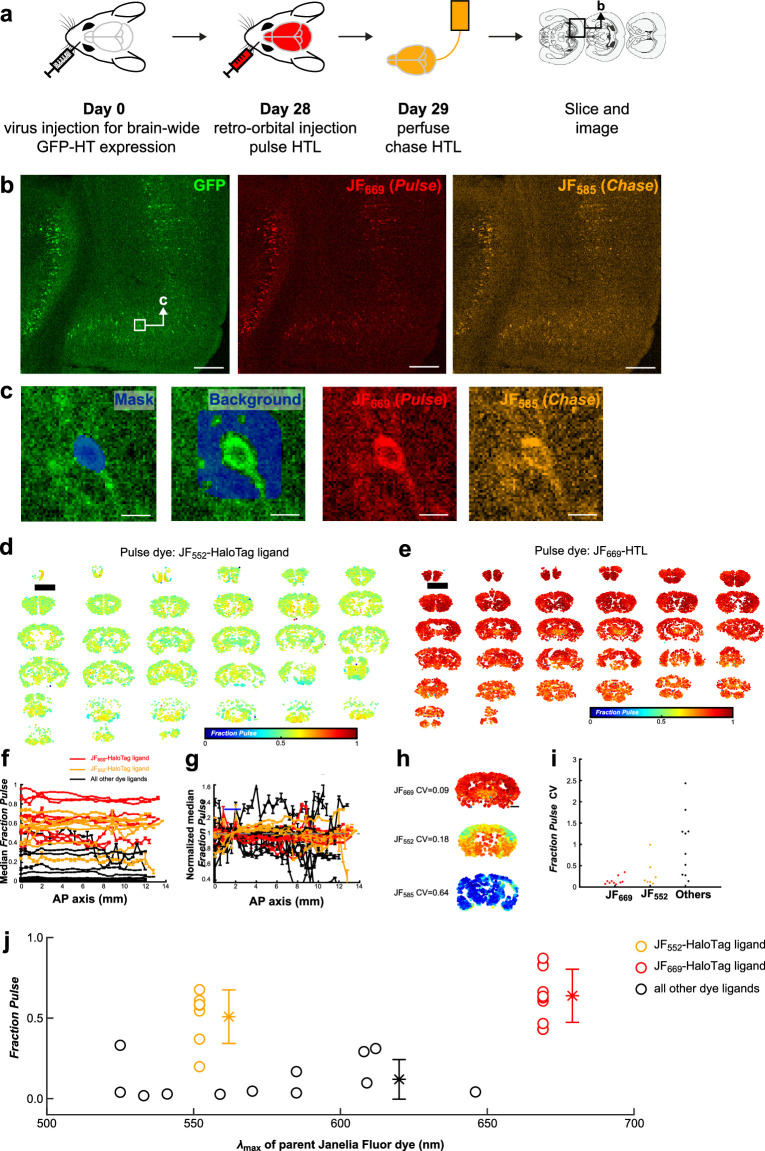
JF_552_ and JF_669_ are bioavailable in the brain. **a**, Experimental procedures for dye-ligand screening *in vivo*. **b**, Example coronal sections imaged 4 weeks after infection with an AAV expressing GFP-HT. GFP fluorescence (left panel), fluorescence of JF_669_-HTL injected *in vivo* (*Pulse*; middle panel), and fluorescence of JF_585_-HTL fluorescence applied during perfusion (*Chase*; right panel). Scale bars, 200 µm. **c**, Example cell (square in b, left panel) overlaid with the mask used to extract signal (first panel), a local background (second panel) and the images of the *in vivo* injected dye-ligand (third panel) and perfused dye-ligand (fourth panel). *Fraction Pulse* is 0.65 in this example. Scale bars are 10 µm. **d**, Coronal slices of GFP-HT injected mouse brain after JF_552_-HTL injections. Each cell is colored by *Fraction Pulse*. The scale bar is 3 mm (black bar in the top left). Notice the uniform labeling across the mouse brain. **e**, same as (d) for JF_669_-HTL. **f**, Median *Fraction Pulse* as a function of AP position (of coronal slices). JF_669_-HTL is in red, JF_552_-HTL is in orange, all other HTL dyes are in black. Error bars are standard errors over the number of cells per slice (medina [5–95]: 743 [58-2604]). **g**, After normalization (mean of the median *Fraction Pulse* under the blue line is set to 1), there is no significant deviation from 1 suggesting uniformity of dye-ligand distribution in this axis (One-way ANOVA, *F*(19) = 1.46, *p* = 0.0913**). h**, For each coronal section of each animal, a coefficient of variation (CV) was calculated. Three examples of coronal sections are shown. **i**, CV of *Fraction Pulse* for JF_669_-HTL injections (Red, CV = 0.15 ± 0.03, *n* = 10), JF_552_-HTL (Orange, CV = 0.31 ± 0.12, *n* = 7) and the other dyes (Black, CV = 2.2 ± 0.81, *n* = 14). **j**, Mean *Fraction Pulse* for each animal (*n* = 31) injected as a function of the dye-ligand excitation wavelength. Here, a higher *Fraction Pulse* signifies better brain bioavailability. JF_669_-HTL (red; *n* = 10, *Fraction Pulse Mean[±SD]*: 0.64 ± 0.16) and JF_552_-HTL (yellow; *n* = 7; *Fraction Pulse Mean[±SD]:* 0.51 ± 0.17) were significantly better than the other dyes (black; *n* = 14, *Fraction Pulse Mean[±SD]*: 0.12 ± 0.12; 1-way ANOVA *F*(2) = 35.96, *p* = 3.2e-08; post-hoc JF_669_ vs. others *p* = 3.8e-8, JF_552_ vs. others *p* = 2.7e-5).

**Extended Data Fig. 4:**
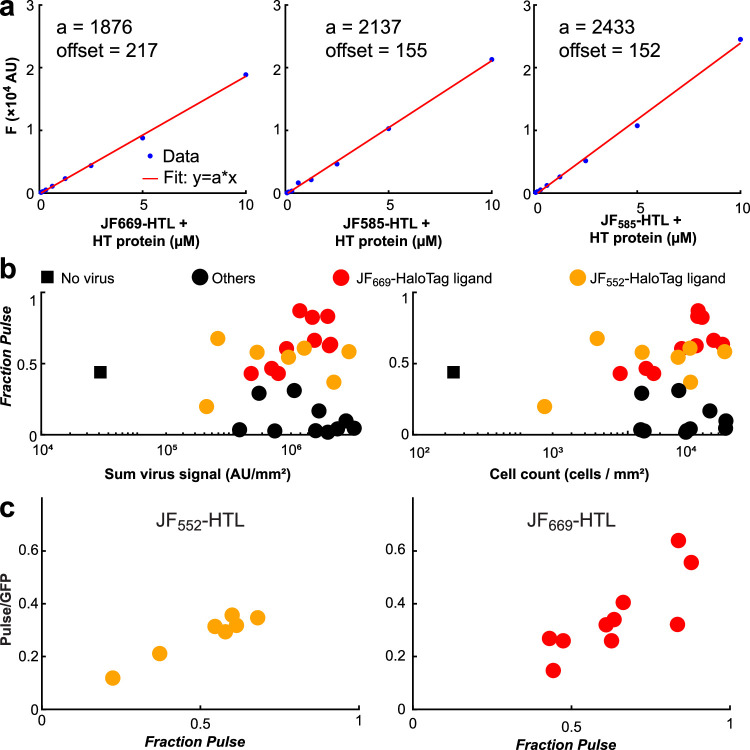
Calibration and validation of GFP-HT based JF dye-ligand screening *in vivo.* **a**, Purified HT was added at saturation to a HTL JF (left: JF_669_-HTL, middle: JF_585_-HTL, right: JF_552_-HTL) in an 8-well coverslip with 20 μL/well at the following concentrations (μM): 10, 5.0, 2.5, 1.25, 0.625, 0.3125, 0.15625, 0. All 8 wells were imaged under the same conditions as the fixed tissue slides (far red channel for JF_669_ and red channel for JF_552_ and JF_585_). The offset at zero was subtracted and is reported in each panel. A linear slope (red line) was fitted to the data (blue circles) without an intercept term (JF_669_-HTL: *r*^2^ = 0.998; JF_585_-HTL: *r*^2^ = 0.9982; JF_552_-HTL *r*^2^ = 0.9963). This calibration covers 20 out of the 30 animals used and a naive calibration was used for the rest of the dyes (150 offset, 2000 slope). **b**, Left, mean *Fraction Pulse* is not correlated with the sum of the virus signal (GFP channel) per animal (Magenta is JF_669_-HTL, red is JF_552_-HTL, black is other dyes; *r*^2^ = 0.02, two-sided Pearson correlation test *p* = 0.45, *n* = 28). An animal that was not injected with a virus (black square) has at least an order of magnitude less summed fluorescence. Right, mean *Fraction Pulse* is not correlated with the number of cells detected per animal (*r*^2^ = 0.005, two-sided Pearson correlation test *p* = 0.717, *n* = 28; normalized by the area of tissue imaged). An animal that was not injected with a virus (black square) has at least an order of magnitude less detected cells**. c**, As the *Fraction Pulse* increases, so is the ratio between the *Pulse* and GFP, indicating that variability in the *Chase* cannot explain the increases in *Fraction Pulse* (Left: JF_552_-HTL: *n* = 7, *r*^2^ = 0.945, two-sided Pearson correlation test *F*(5) = 85.5, *p* = 0.00025; Right: JF_669_-HTL: *n* = 10, *r*^2^ = 0.65, two-sided Pearson correlation test *F*(8) = 14.7, *p* = 0.00495). **X-axis**: *Fraction Pulse* [*Pulse* / (*Chase* + *Pulse*)]. This compares the pulse injected *in vivo* to the total protein calculated by using the two dyes with their calibration. **Y-axis**: *Pulse to GFP ratio*. This compares the pulse injected *in vivo* to the total protein as estimated by the GFP fluorescence. The fact that these 2 ways correlate, means that we are obtaining reliable estimates for the dye-ligand calibrations (needed to calculate *Pulse* + *Chase*).

**Extended Data Fig. 5:**
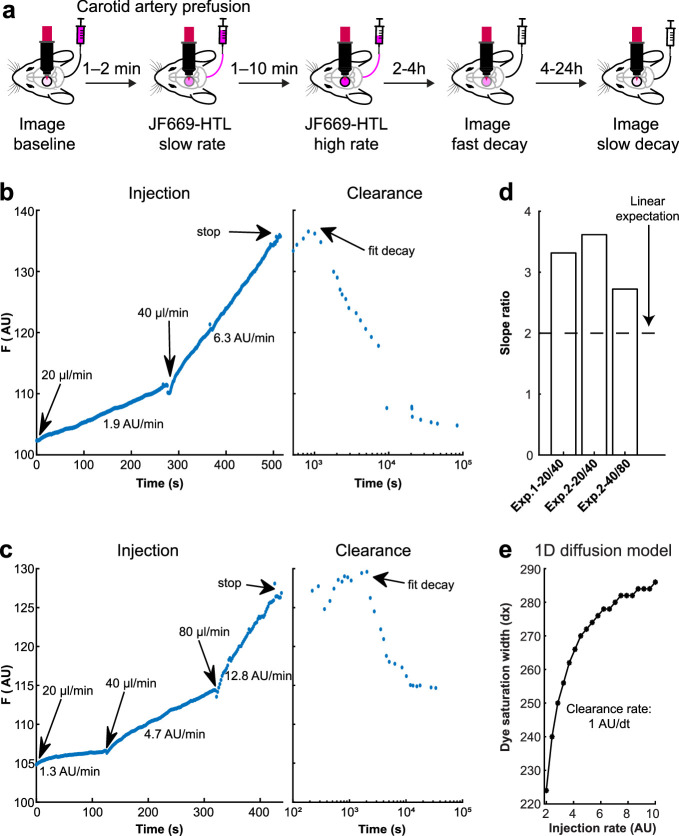
Injection pharmacokinetics of JF dyes. **a**, Design of experiment to measure dye-ligand injection pharmacokinetics. Continuous imaging was performed during carotid artery perfusion of JF_669_-HTL at different rates. **b**, One animal, using infusion rates of 20 µl/min and 40 µl/min. **c**, Another animal, using 20, 40, and 80 µl/min. **d**, Slope ratios for all transitions in infusion rates (two animals, three transitions). If clearance was proportional to the amount of dye-ligand injected, a slope ratio of 2 is expected. The slope ratio of > 2 implies sublinear clearance or saturation of clearance mechanisms. **e**, 1D diffusion model shows an increase in the area of saturation (y-axis) with increasing dye-ligand injection rate (x-axis). See Supplementary Text for further details.

**Extended Data Fig. 6:**
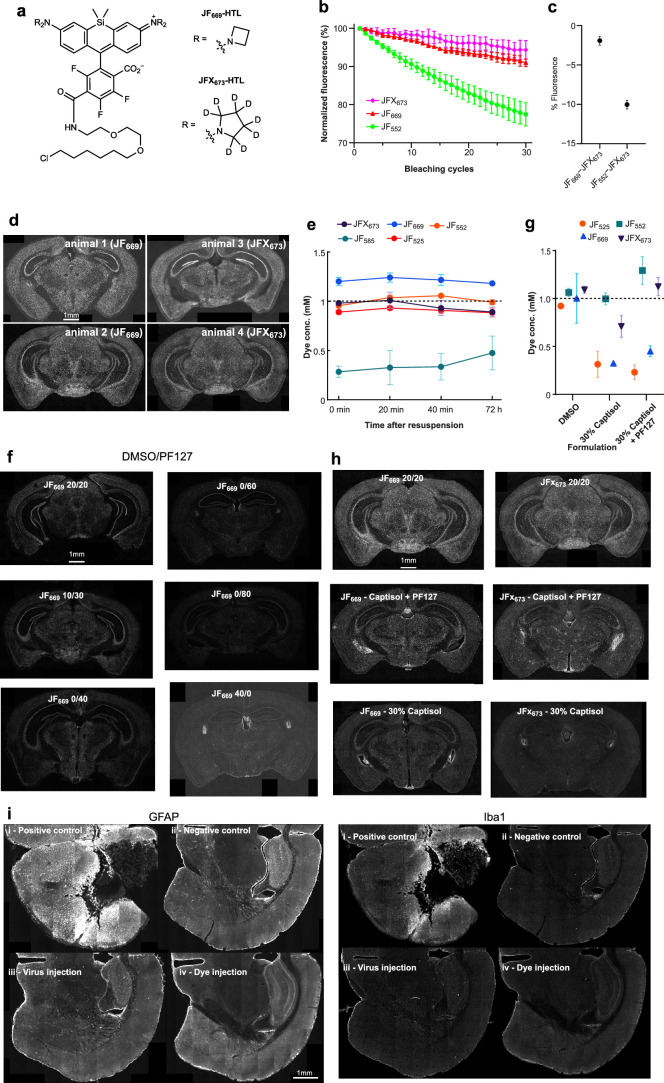
Validation of dye-ligand formulation and JFX_673_-HTL, a bioavailable red dye-ligand with improved photostability. **a**, Comparison of JF_669_-HTL and JFX_673_-HTL. Note the addition of deuterium and additional carbon in the R position of JFX_673_. **b**, Bleaching curves of JF dyes showing normalized fluorescence over 30 bleaching cycles. Plotting mean and SD for n = 3 for each dye-ligand / timepoint **c**, JFX_673_-HTL was significantly more photostable than the other dyes tested. Plotting mean and SD for n = 3 for each dye-ligand **d**, Example sections of MeCP2-HT mice do not show notable differences in brain availability of JF_669_-HTL versus JFX_673_-HTL. All panels show the pulse dye. **e**, Solubility of JF-HTL dyes using DMSO (20 μl), Pluronic F127 (20 μl), and PBS (60 μl) formulation over time after resuspension. No significant decrease in solubility was observed and all *in vivo* dyes used reached the intended solubility of 1 mM **(**2-way ANOVA [Dye-ligand x Time] p < 0.05; Time: *F*(3,20) = 0.66, *p* = 0.5835; Dye: *F*(4,20) = 149.21, p < 0.0001; Interaction: *F*(12,20) = 1, *p* = 0.4794). Plotting mean and SD for n = 3 for each dye-ligand / formulation. **f**, Example coronal slices from MeCP2-HaloTag animals injected with different ratios of DMSO and Pluronic F127. The original 1:1 ratio was the best, as shown by the brighter pulse dye-ligand staining. **g**, Different formulation than (e) to test the replacement of DMSO (left) with Captisol (middle) or a combination of Captisol and Pluronic F127 (right). We saw mixed effects on solubility, while some dyes (JFX_673_-HTL) retained solubility, and some did not (JF_669_-HTL; 2-way ANOVA, interaction: *F*(6,24) = 13.31, p 1.61e-6; Dye: *F*(3,24) = 67.35., *p* < 0.0001; Formulation: *F*(2,24) = 46.91, *p* = *1e-9*). Plotting mean and SD for n = 3 for each dye-ligand / timepoint **h**, Example coronal slices from MeCP2-HaloTag animals injected with solutions from (f). Both dyes that were less soluble with Captisol alone or Captisol with Pluronic F127 (JF_669_ left) or those that were as soluble (JFX_673_ right) were less bioavailable without DMSO. n = 1 for each condition. **i**, Immunohistochemistry against GFAP (left) and Iba1(right) for 4 conditions, i-Positive control: A cortical lesioned animal. ii-Negative control: Naive animal without any manipulation. iii-Virus injection: An animal was injected with the GFP-HaloTag virus and perfused 3 weeks after injection. iv-Dye-ligand injection: An animal was injected with JF_669_-HTL and perfused 24 h after injection. Both dye-ligand injection and virus injection do not increase GFAP or Iba1 levels. n = 1 for each condition.

The DELTA protocol requires fluorescent HTL dyes that are spectrally separable and bind HT fusion proteins efficiently in vivo. A screen of JF-HTL dyes^33–36^ in mouse brains identified JF_669_-HTL and JF_552_-HTL as particularly bioavailable, with both HTL dyes having strong and uniform labeling across the brain (Extended Data Figs. 3 and 4 and Supplementary Text). We subsequently tested a more photostable dye, JFX_673_-HTL^39^, which retained the high bioavailability of its parent JF_669_-HTL^34^. Finally, we investigated the optimal in vivo dye ligand formulation and delivery protocols (Methods, Extended Data Figs. 5 and 6 and Supplementary Text).

**Extended Data Fig. 7:**
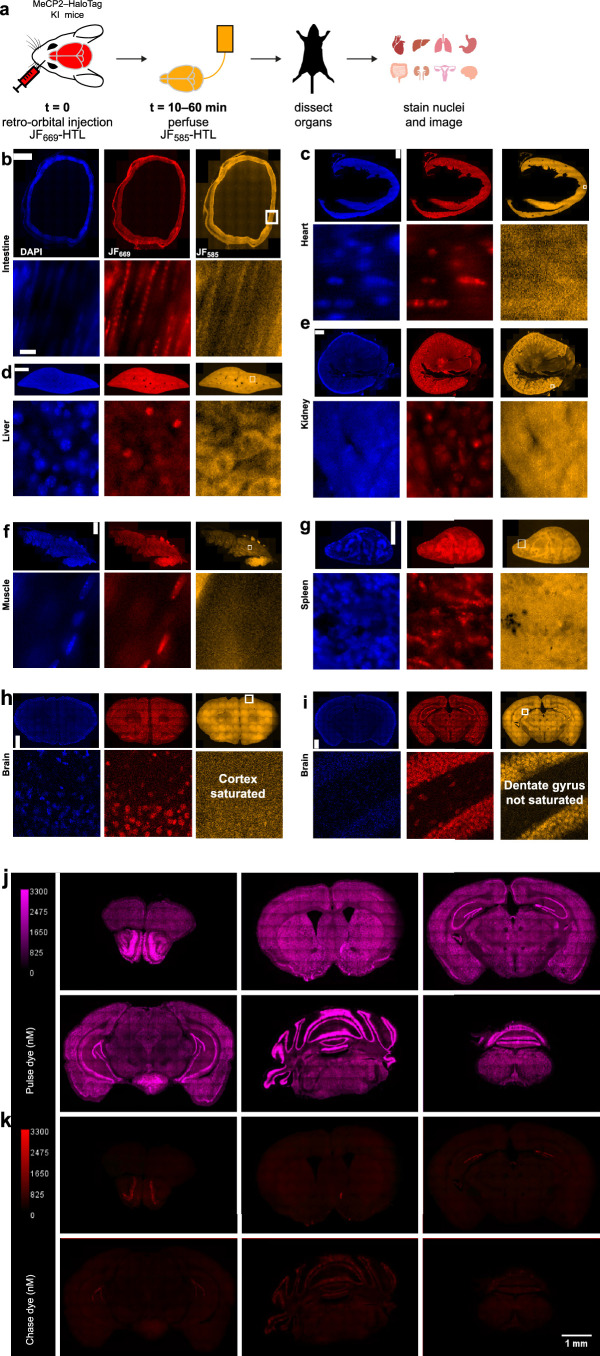
Systemic injection of ligand dye-ligand saturates the abundant protein MeCP2-HT in most organs. **a**, Schematic of the experimental procedures to test the saturation of MeCP2-HT. **b-i**, Organs harvested and imaged for pulse dye-ligand saturation (red), as evident by the lack of chase dye-ligand (orange) in the nuclei (blue) where MeCP2-HT is expressed. Top scale bar is 1 mm, bottom scales bars are 50 μm. **j-k**, Six coronal sections of an MeCP2-HT animal injected *in vivo* with JF_669_-HTL (j) immediately followed by perfusion with JF_552_-HTL (k) to check for saturation. All panels were converted to nM dye-ligand concentration and scaled to the same concentration. Only very dense cell body regions, highly enriched in MeCP2 (*for example*, i), show any chase dye-ligand staining, indicating the inability to saturate *in vivo*. n = 5 animals for the brain, n = 1 for the other organs.

**Extended Data Fig. 8:**
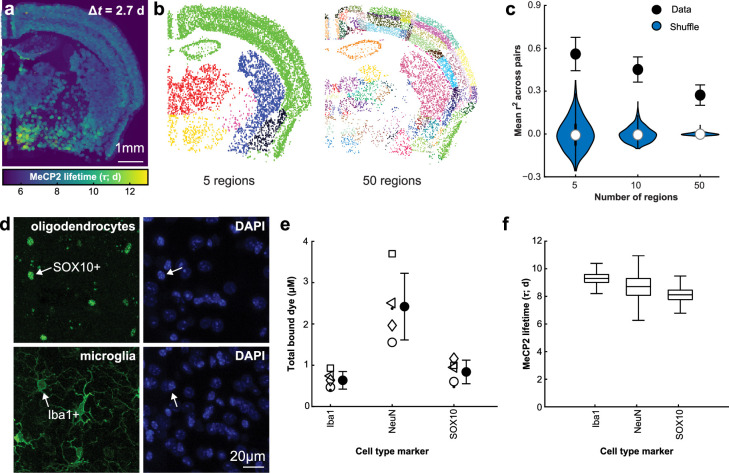
The measured lifetime of MeCP2-HT is consistent across individual mice and cell-types. **a**, Example coronal section of the lifetime of MeCP2-HT after segmentation of NeuN positive nuclei. Same data as Fig. 1c-f n = 5 animals **b**, Example assignment of neuronal nuclei to brain regions based on the Allen CCF v3 at different levels (left 5 regions, right 50 regions). **c**, Protein lifetime measurements were consistent between mice as assessed by examining correlations between all possible pairs of animals (*n* = 5 animals, 10 pairs). For three levels of the CCF (5, 10, and 50 regions) the correlation between regions was higher than for a shuffled control. **d**, Two additional sections were stained in each of the five animals for oligodendrocytes (SOX10, top) and microglia (Iba1, bottom). **e**, MeCP2 was more abundant in the nuclei of neurons (Iba1: 0.63 ± 0.21, NeuN: 2.42 ± 0.8 SOX10: 0.84 ± 0.28 µM total dye-ligand signal; two-sided ANOVA *F*(2) = 27.22, *p* = 2e-4).Plotting mean and SD for n = 5 – individual symbols. **f**, Lifetimes were similar for all cell types, as confidence intervals overlapped between all cell types (median and [5th-95th percentiles] of: Iba1: 9.3 [8.4-10.1], NeuN: 8.7 [6.9-10.3], SOX10: 8.1 [7.1-9.1] days of MeCP2 lifetime). Plotted are median with 25-75 percentile as the box and 5–95 CI as the whiskers from n = 3000 bootstrap iterations.

We used DELTA to measure the lifetime of the nuclear protein methyl-CpG binding protein 2 (MeCP2)^40–42^ using MeCP2–HT knock-in mice^43^. As MeCP2 is relatively abundant (top 10% in the brain^44^), achieving dye ligand excess would signify DELTA’s broad applicability (Extended Data Fig. 7). Previous MeCP2 lifetime measurements using MS also provide a point of comparison for our method^10^. We compiled multiple estimates of protein lifetime from the same animal by sequentially injecting the highly bioavailable dye ligands JF_669_-HTL and JF_552_-HTL, followed by perfusion of a third dye ligand: JF_608_-HTL. After perfusion, the brain was sectioned and stained with DAPI to identify all nuclei, and immunofluorescence (IF) was used to classify cell types (Fig. 1c and Extended Data Fig. 8d). We imaged all three dyes at subcellular resolution and calculated the fraction pulse for all neuronal nuclei across the brain of five MeCP2–HT mice. We converted the measurements to average protein lifetime (*τ*; Fig. 1d,e). The lifetime of neuronal MeCP2 differed across brain regions (medians ranged from 9.2 to 15.2 days; Fig. 1f and Extended Data Fig. 8a–c). The longest MeCP2 lifetime was in the cerebellum (Fig. 1e), in agreement with previous measurements using MS^10^. Also consistent with previous studies, we found that MeCP2 was expressed at higher levels in neuronal cells than in glia (Extended Data Fig. 8d–f)^45^. These results show that DELTA is suitable for probing the lifetimes of abundant proteins at brain-wide scales.

### Environmental enrichment alters PSD-95–HT dynamics

**Fig. 2:**
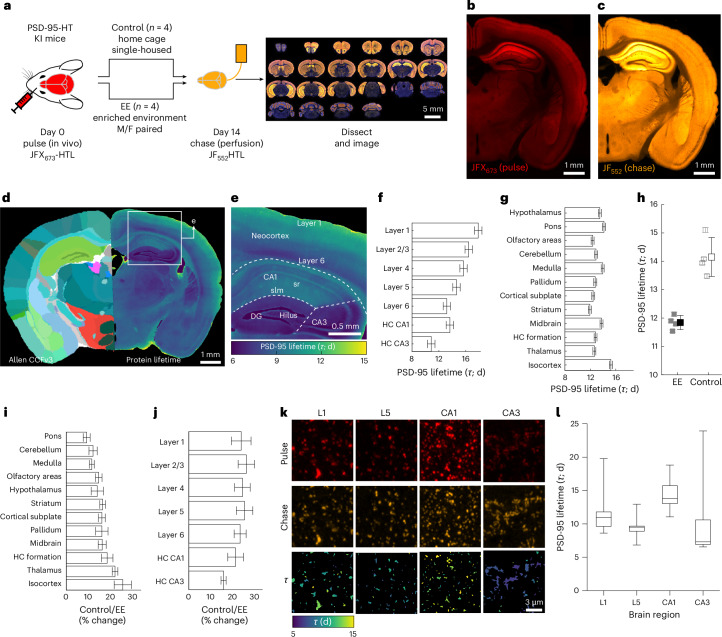
PSD-95 turnover depends on experience. **a**, Experimental design for turnover measurement in a PSD-95–HT knock-in mouse. **b**–**e**, Example coronal sections showing the pulse (**b**), chase (**c**) and calculated lifetime aligned to the Allen CCFv3 (**d** and **e**). Note the lifetime gradient that separates the CA1 stratum radiatum (sr) and stratum lacunosum moleculare (slm; long lifetime) from dentate gyrus (DG) and hilus (all shorter lifetimes). **f**, Average ± s.e. lifetimes of control animals (*n* = 4). Cortical layers and subfields of the hippocampus (HC) were significantly different (mixed-effects linear model with layers as fixed effects (means: 10.0–16.5 days, s.e.: ~0.5, all *P* < 0.0001) and animal ID as a random effect (mean, 1.42 days; residual error, 1.39 days)). All mixed-effects models are double sided and without multiple-comparison corrections. **g**, Average ± s.e. lifetimes for 12 large brain regions were also significantly different in control mice (*n* = 4; mixed-effects linear model with brain regions as fixed effects (means: 11.9–15.2 days, s.e.: ~0.23, all *P* < 0.0001); animal ID as a random effect (mean: 0.46 days); residual error: 1.8 days). **h**, Average ± s.e. lifetimes of four mice under EE and four mice under control conditions. EE increased protein turnover and shortened the average lifetime of PSD-95–HT. Individual animals in gray (EE: 11.8 ± 0.2 days, *n* = 4; control: 14.2 ± 0.7 days, *n* = 4; two-sided Wilcoxon rank-sum test *W* = 26, *P* = 0.0286). **i**, Percentage change ± s.e. in control versus EE animals for 12 different brain regions; Mixed-effects linear model for each brain region with group assignment as fixed effects and animal as a random effect. **j**, Same as **i** for cortical layers and HC subfields. **k**, Example images using Airyscan imaging of ExM tissue (maximum projection of five *z*-planes, 0.3 μm apart) from layer 1, HC CA1 subfield and basal dendrites of CA3 showing both pulse, chase and lifetime (*τ*). **l**, Quantification of segmented single-synapse turnover (median lifetime in days; L1, 10.97; L5, 9.5; CA1, 13.78; CA3, 7.32). Boxes show the interquartile range and whiskers mark the 5th and 95th percentiles.

**Extended Data Fig. 9:**
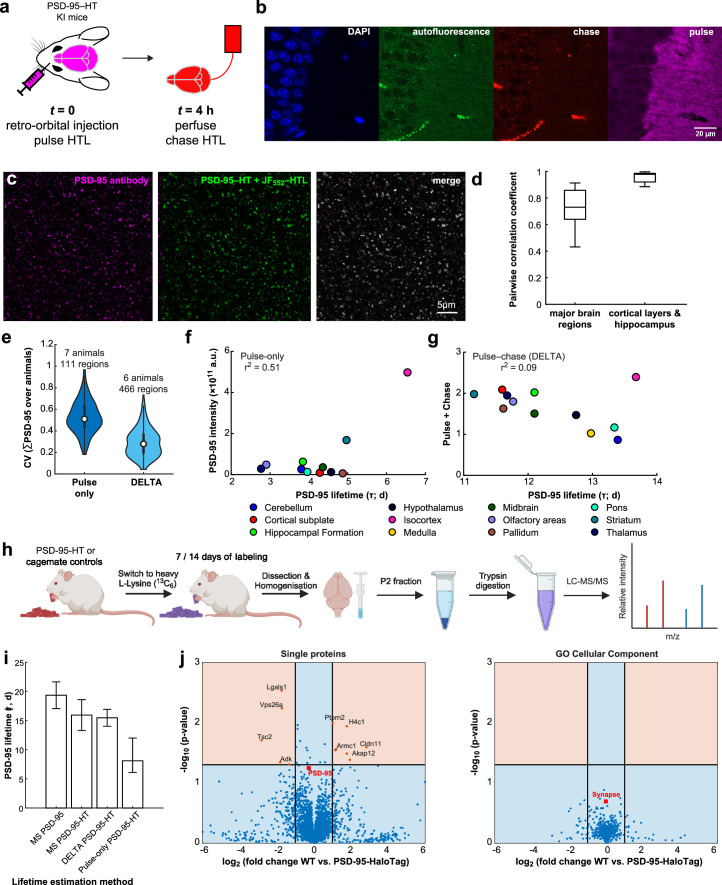
Validation of DELTA in the PSD-95-HaloTag mouse. **a**, Experimental procedures for testing saturation in a PSD-95–HT mouse. **b** Example section showing the basal dendrite section of the CA1 region of the hippocampus (DAPI shows the nuclei in the leftmost panel). The green channel (autofluorescence; 2^nd^ panel from left) accounts for all the signal in the red channel (*Chase* – JF_552_-HTL; 3^rd^ panel), while the far-red channel shows the expected pattern of synapses (*Pulse* – JFX_673_-HTL; 4^th^ panel). These results were consistnat across n = 3 mice tested. **c**, Validation of HTL signal (JF_552_-HTL; middle panel) in the PSD-95-HT knock-in animal with a knockout validated antibody (left panel) showing high colocalization (right panel). Repeated for 4 fields of view in 1 animal. **d**, Box plot of all pairs of animals (6 animals, *n* = 15 pairs) for correlation coefficients for the lifetime of PSD-95 across 12 large brain regions (left panel; As in Fig. 2g, i) or cortical layers and HC subfields (right; As in Fig. 2f, j). Shown are the median (center), interquartile range (bounds of the box), and whiskers representing 1.5× the interquartile range. **e**, Violin plot of coefficient of variation (CV) across animals for the total PSD-95 measured for each brain region. Pulse only is publicly available data from Bulovaite et al.^29^ where we used the standard deviation divided by the mean of integrated fluorescence at day 0 (*n* = 7 animals; *n* = 111 brain regions). For DELTA we used the standard deviation divided by the mean of the total *Pulse* and *Chase* values (*n* = 6 animals; *n* = 466 brain regions). Measurement variability of total PSD-95 across animals was lower in DELTA then in Bulovaite et al. (two-sided Wilcoxon rank sum test; *z* = 12.6; *p* = 1.3e-36). Shown are also the median (circle), interquartile range (bounds of the box), and whiskers representing 1.5× the interquartile range. **f-g**, Correlation between estimated expression of PSD-95 (y axis) with its lifetime measurement (x axis) from data of Bulovaite et al. (f; Pulse only) or from DELTA (g) for twelve brain regions (colored circles). r^2^ values from linear regression are shown for each panel. See Supplementary Text for details. **h**, Workflow for mass spectrometry (MS) measurements of protein turnover. **i**, Comparisons of PSD-95 mean lifetime with or without HaloTag fusion by different methods. There is an agreement between DELTA and MS based measurements of lifetime for PSD-95-HT. The non-overlapping confidence intervals of the pulse-only approach signifies a statistically significant difference. n = 4 mice for MS; Same data as Fig. 2g for DELTA; Pulse only data from ref# 29. **j**, Volcano plots comparing MS-based proteome-wide lifetime estimates between PSD-95-HaloTag knock-in mice and WT cage mates. Left, per protein analysis showing most proteins don’t significantly change in lifetime (y-axis: -log_10_ *p* value – two-sided; horizontal line at *p* = 0.05; x-axis: log_2_ fold change; vertical lines at two-fold increase or decrease). PSD-95 is highlighted with a red square; significant single proteins are labeled with their gene names. Right, same as left but averaging proteins based on their GO Cellular Component annotations. Synapse Cellular Component is highlighted with a red square. No correction was made for multiple comparisons.

PSD-95 is an abundant scaffold protein in the postsynaptic density of excitatory synapses^46^ and anchors glutamate receptors, channels and synaptic cell adhesion molecules in synapses^47^. Synaptic PSD-95 content is known to be regulated by protein synthesis and degradation^48,49^. We measured brain-wide PSD-95 dynamics and the effects of behavioral manipulations in synapses using knock-in mice expressing PSD-95 fused with HaloTag (PSD-95–HT^50^; Extended Data Fig. 9). Mice housed in standard cages (*n* = 4) and mice placed in an enriched environment^51,52^ (EE; *n* = 4) were imaged with a pulse–chase interval of 14 days, using JFX_673_-HTL as the pulse injected in vivo and JF_552_-HTL as the chase in perfusion (Fig. 2a). This order of dye ligands ensures saturation of all expressed proteins due to the high bioavailability of JFX_673_-HTL, which is similar to its parent dye ligand, JF_669_-HTL (Extended Data Figs. 3j and 6d). Following imaging (Fig. 2b,c), sections were registered to the Allen Brain Reference Atlas (CCFv3)^53^, and the lifetime of PSD-95–HT was calculated (Fig. 2d,e). PSD-95–HT lifetimes in control animals varied by 50% across brain regions and cortical layers (range, 10.9–17.8 days; Fig. 2f,g). The brain-wide average estimated lifetime of PSD-95 was more than 2 days (~19%) shorter in mice housed for 2 weeks in EE compared to control (Fig. 2h). The neocortex showed the largest reduction in PSD-95 lifetime with EE (~25%; Fig. 2i). The effects of EE on lifetime were similar across cortical layers but differed in hippocampal subfields, with CA1 affected more than CA3 (Fig. 2j).

We detected a small (up to 20%) increase in total PSD-95–HT level in some brain regions after EE, disproportionately caused by an increase in chase (that is, newly synthesized protein) that were not counterbalanced by a decrease in pulse (that is, degradation of protein). The additional newly synthesized protein transiently decreases protein lifetime of PSD-95–HT, but the effects on protein lifetime estimates are small (approximately 5%) and transient (Methods and Extended Data Fig. 2i).

PSD-95 lifetimes measured by the pulse–chase DELTA were substantially longer than those measured previously using HTLs in a pulse-only approach^29^ (for example, for control neocortex, mean (95% confidence interval (CI)): 15.2 (14.7–15.6) days for DELTA versus 8.1 (6.1–12.0) days for pulse-only), even though both methods are based on the same PSD-95–HT knock-in mice (Extended Data Fig. 9f,g). To obtain an independent estimate, we turned to heavy stable-isotope-based MS measurements of protein turnover. We fed either wild-type (WT) or homozygous PSD-95–HT knock-in mice a ^13^C_6_-lysine diet for 7 or 14 days (Extended Data Fig. 9h; four animals per group for each pulse interval; *n* = 16 animals in total). The neocortex was then isolated and protein lifetime was measured using standard methods^10^. We detected a small difference in the lifetime of PSD-95 in WT versus HT knock-in mice (mean (95% CI): WT: 19.3 (17.1–21.6) days, HT knock-in: 15.9 (13.3–18.6) days, *t*-test *t*(8) = 2.2268, *P* = 0.056, *n* = 4 for each). The MS measurements showed turnover rates close to those measured by DELTA (15.2 (14.7–15.6) days), but longer than estimates using the pulse-only method^29^ (Extended Data Fig. 9i). The MS measurements also showed that turnover rates were unchanged for most other proteins in the PSD-95–HT knock-in compared to WT animals (Extended Data Fig. 9j and Supplementary Table 3).

Resolving DELTA-based lifetime measurements of PSD-95-HT at the level of individual synapses requires approximately fourfold higher imaging resolution than permitted by the diffraction limit^54^. We developed an expansion microscopy (ExM) protocol that enables twofold expansion without proteolysis or strong protein denaturation, thus fully retaining HT-bound JF-HTL dyes. Imaging with Airyscan confocal microscopy^55^ provided another twofold improvement in resolution, providing the required fourfold enhancement. Combined with our use of bright and photostable small-molecule dyes, this approach enabled measurements of turnover at individual synapses. We measured the turnover in individual synapses of neocortical layers 1 and 5, and hippocampal subfields CA1 and CA3 (Fig. 2k,l, Extended Data Fig. 9c and Supplementary Movie 1). Our analysis revealed that layer 1 exhibited slower turnover compared to layer 5 in the neocortex and, similarly, CA1 showed slower turnover than CA3 in the hippocampus. While these trends align with those observed in whole-brain imaging, it is important to note that our single-synapse imaging focuses on a smaller, localized region within each structure. As a result, these localized measurements are not directly comparable to whole-brain imaging, which involves averaging across cellular compartments.

These results demonstrate that DELTA can accurately estimate synaptic protein lifetimes with brain-wide coverage, up to single-synapse resolution, and is sensitive to behavioral manipulations.

#### Learning increases GluA2–HT turnover in the hippocampus

**Extended Data Fig. 10:**
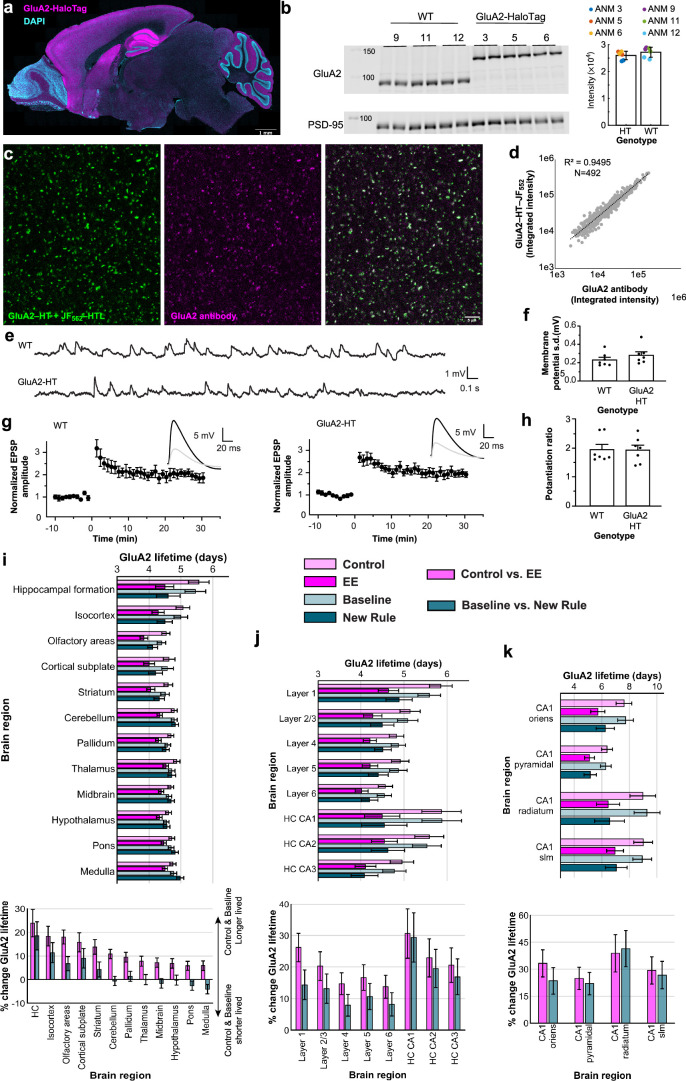
GluA2-HaloTag validation for use in DELTA brain-wide. **a**, Sagittal section for a GluA2-HT knock-in mouse perfused with JFX_673_-HTL showing the distribution of GluA2 (magenta). Nuclei were stained with DAPI (white). Consistent results were seen in 3 animals. **b**, Left, Western blots stained for GluA2 and PSD-95 as normalization. 12 lanes were used: 3 WT animals (Lanes marked: 9,11,12) and 3 homozygous GluA2-HT knock-ins (Lanes marked: 3,5,6) both done in duplicates. Right, quantification of GluA2 signal normalized to PSD-95 averaged across animals and replicates for WT (27,186 ± 2004) and GluA2-HT knock-ins (25,963 ± 1685). **c**, Validation of HTL signal (JF_552_-HTL; LEFT panel) in the GluA2-HT knock-in animal with an antibody (middle panel) showing high colocalization (right panel). Consistnat results were seen in 4 fields of view from 1 animal. **d**, Segmentation of 492 synapses from (c) quantifying the integrated intensity in the HTL-dye channel (y axis, log scale) vs. the antibody signal (x-axis, log scale) showing very high correlation (r^2^ = 0.9495) **e**, Example spontaneous activity traces of WT (top) and knock-in (bottom) from acute HC slices. **f**, Quantification of spontaneous activity as the standard deviation of the traces show no significant difference (WT: 0.23 ± 0.03 mV, knock-in: 0.28 ± 0.04 mean ± SE, *n* = 7 cells for both, *p* = 0.29 2-sided T-Test). **g**, Example LTP induction in WT (top) and knock-in (bottom). **h**, Quantification of the potentiation ratio shows normal LTP of the knock-in compared to WT (WT: 1.96 ± 0.17 knock-in: 1.98 ± 0.18 mean ± SE, *n* = 7 cells for both, *p* = 0.94 2-sided t-test). **i-k**, Average lifetimes (top) of the 4 behavioral conditions (Control, EE, Baseline, New Rule) and 2 comparisons of lifetime (bottom; Control vs. EE and Baseline vs. New Rule) for the 12 large brain regions (i) neocortical layers and HC subfields (j) and for CA1 lamina (k). All statistics in i-k come from a Mixed Effects model in which group assignment is a fixed effect and animal IDs are random effects. Baseline n = 4 mice; New Rule n = 3 mice; Control n = 4 mice; EE n = 5 mice. Source data

We next used DELTA to identify brain regions with enhanced synaptic plasticity during learning. As ionotropic glutamate receptors, including GluA2, directly influence synaptic strength and are modified during synaptic plasticity^56–58^, we probed turnover in a novel GluA2–HT knock-in mice (Extended Data Fig. 10a). Homozygous GluA2–HT mice had normal GluA2 protein levels (Extended Data Fig. 10b) and showed high correlation between the HTL-dye signal and immunostaining of GluA2 (Extended Data Fig. 10c,d). Recordings from CA1 pyramidal neurons in acute hippocampal brain slices showed normal synaptic transmission (Extended Data Fig. 10e,f) and long-term potentiation (Extended Data Fig. 10g,h).

**Fig. 3:**
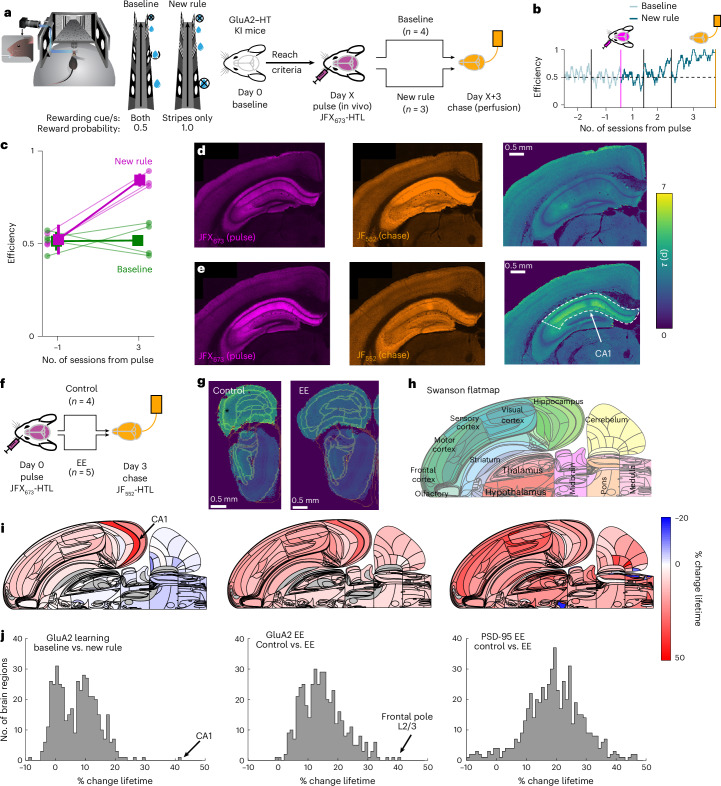
Learning-induced GluA2 turnover is more localized than following environmental enrichment. **a**, Turnover measurement in a GluA2–HT knock-in mouse during learning of a new behavioral rule (new rule). **b**, Example experiment showing efficiency (number of rewards per number of cues licked) across five daily sessions. Turnover was assessed by pulse dye ligand (JFX_673_-HTL) injection before new-rule and chase dye ligand (JF_552_-HTL) perfusion after the final session. **c**, Behavioral efficiency comparison between baseline (green, unchanged) and new-rule (magenta, improved) groups (*n* = 7). **d**,**e**, Coronal sections showing pulse (magenta), chase (orange) and calculated GluA2–HT lifetime (fast turnover, green and blue; slower turnover, yellow) for new-rule (**d**) and baseline (**e**) conditions, highlighting CA1 differences. Example from (**c**) *n* = 7 animals. **f**, Experimental design for turnover measurement of GluA2–HT modulated by environmental enrichment (EE). **g**, Example coronal sections from control mice and after EE. A black asterisk indicates the lower lifetime of GluA2–HT in frontal cortical regions. Example from *n* = 9 animals. Color scale is the same as in **d**. **h**, Swanson flatmap^73^ representation of mouse brain regions. Different colors represent different brain regions. **i**, Left: percentage change in GluA2–HT lifetime in baseline versus new rule. The largest effect is in CA1 (arrow). Middle: control versus EE groups. The largest effect is in the frontal pole (arrow). Right: percentage change in PSD-95–HT lifetime in control versus EE groups. **j**, Distribution of turnover changes across conditions. GluA2 learning (*n* = 442, median = 7.5%, 59.5% of brain regions with >5% change); GluA2 EE (*n* = 442, median = 14.3%, 94.8% >5%); PSD-95 EE (*n* = 580, median = 19.2%, 95.3% > 5%).

To study the turnover of synaptic proteins associated with learning, head-restrained, water-restricted mice were trained in a virtual reality environment. The mice were conditioned to lick for water rewards as visual cues appeared at pseudorandom locations within an infinitely extending corridor (Methods and Fig. 3a). We first trained mice to gather rewards at locations marked with one of two distinct visual cues (stripes and circles; reward probability 50% for both cues). After mice (*n* = 7) reliably licked in the presence of visual cues, but not otherwise, they were injected with the pulse dye ligand (JFX_673_-HTL). To investigate new learning, a subset of mice was switched to a task variant in which only the striped visual cue was rewarding while the other cue was not (new rule; *n* = 3; striped reward probability, 100%). Mice learned to lick only in the presence of the striped visual cue, as indicated by increased licking efficiency within 3 days (efficiency = number of rewards per number of cues licked; see an example mouse in Fig. 3b). The other mice (baseline; *n* = 4) remained on the initial task and maintained licking efficiency around 50%. At the end of 3 days with one behavioral session per day, there was a clear difference in licking efficiency between the groups, and all mice were perfused with the chase dye ligand (JF_552_-HTL; Fig. 3c).

Brains were imaged and aligned to the Allen Brain Reference Atlas (CCFv3; Fig. 3d,e). In the raw data, large changes in GluA2–HT turnover were apparent in the neocortex and hippocampus. The new-rule group CA1 region had low pulse signal (Fig. 3d (left panel)) and high chase signal (Fig. 3d (middle panel)), indicating relatively fast GluA2–HT turnover (Fig. 3d (right panel)). In the baseline group, CA1 and superficial neocortical layers showed higher pulse signal (Fig. 3e (left panel)) and lower chase signal (Fig. 3e (middle panel)) indicating slower GluA2–HT turnover (Fig. 3e (right panel)). Neither group showed any change in total protein.

We compared the changes in GluA2–HT turnover during learning versus EE (Fig. 3f and Extended Data Fig. 10i–k; control *n* = 4; EE *n* = 5). EE accelerated turnover of GluA2–HT, but, unlike rule learning, the largest effect of EE was in frontal cortical areas (~40% in layer 2/3 of the frontal pole and nearby regions of the secondary motor cortex and orbital cortex; Fig. 3g). We compared all three manipulations using the Swanson flatmap representation (Fig. 3h). The largest difference in GluA2–HT turnover in the new-rule group was in the hippocampus (Fig. 3i (left panel)). Changes caused by EE both for GluA2–HT (Fig. 3i (middle panel)) and for PSD-95–HT (Fig. 3i (right panel)) were more widely distributed across brain regions. Unlike PSD-95–HT, total GluA2–HT levels were stable across all four conditions (linear mixed-effects (LME) model: no significant effect of group assignment as compared to the control group; *P* = 0.831, *P* = 0.973 and *P* = 0.995 for EE, baseline and new-rule groups, respectively).

We examined the similarity of plasticity across conditions by characterizing the distributions of turnover changes. All three distributions were different (Kruskal–Wallis test *χ*^2^ = 408, *P* = 2.1 × 10^−89^, Tukey–Kramer post hoc test, all medians were significantly different) with GluA2 learning showing the smallest median (Fig. 3j (left panel), *n* = 442, median = 7.5%) followed by GluA2 EE (Fig. 3j (middle panel), *n* = 442, median = 14.3%) and PSD-95 EE showing the largest median (Fig. 3j (right panel), *n* = 580, median = 19.2%). For EE in both PSD-95 and GluA2, the distribution shows most brain regions were modulated (95.3% and 94.8% of brain regions with over 5% change), whereas for learning in GluA2 the distribution was sparser with more brain regions centered around 0% change (59.5% of brain regions with over 5% change). These data show that the distribution of turnover varies across synaptic proteins, even in the same complex (that is, synapses), and across different behavioral manipulations.

These results indicate that DELTA can be used to identify the loci of plasticity for multiple synaptic proteins and behavioral manipulations.

#### Subcellular regulation of synaptic protein turnover

**Fig. 4:**
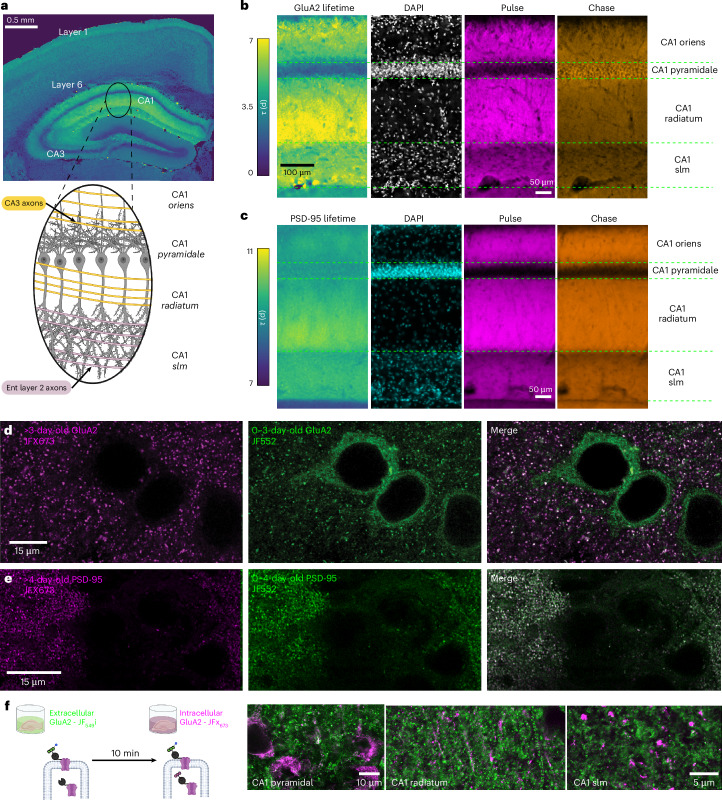
Subcellular regulation of synaptic protein turnover. **a**, Top: image of GluA2–HT lifetime in a coronal section. Bottom: schematic of the orientation of pyramidal cells in CA1 (gray) with inputs from CA3 (yellow) and entorhinal cortex (pink). Similar lifetime gradients were observed in all animals regardless of condition (*n* = 16). **b**, Lifetimes are low in the soma (stratum pyramidale), and increase in the proximal dendrites in stratum oriens and radiatum and decrease in the stratum lacunosum moleculare, implying subcellular control of synaptic protein turnover. **c**, Same as **b** for PSD-95–HT, showing similar turnover trends. **d**,**e**, Differences between GluA2–HT (**d**) and PSD-95–HT (**e**) in subcellular localization of newly synthesized protein. Whereas >3-day-old protein (magenta; left images) is mostly synaptically localized in both GluA2–HT (**d**) and PSD-95–HT (**e**), newly synthesized GluA2 (green; middle image in **d**) is enriched around the soma (in the cytosol while excluded from the nucleus; Supplementary Movie 2), but PSD-95–HT is not (green; middle image in **e**). Merged view in the right images. Similar gradients were observed in all animals regardless of condition (GluA2–HT, *n* = 16; PSD-95–HT, *n* = 8). **f**, Left: illustration of live slice experiment that separates extracellular and intracellular pools of GluA2–HT. The extracellular pool is first labeled with a cell-impermeable dye ligand (JF_549_i-HTL, green) followed by labeling with a cell-permeable dye ligand (JFX_673_-HTL, magenta). Both pools are seen in somata (first image) and both stratum radiatum and stratum lacunosum moleculare dendrites (second and third images; four brain slices).

Synaptic protein turnover was not uniform across the dendritic arbor, hinting at compartment-specific plasticity. This was most apparent in the CA1 region of the hippocampus, where cell bodies reside in a narrow layer (Fig. 4a; stratum pyramidale) and axons from different pathways impinge on dendrites of the same cells in different layers. CA3 axons arrive on proximal dendrites (basal, stratum oriens; apical, stratum radiatum), whereas axons from layer 3 of entorhinal cortex innervate distal apical dendrites (stratum lacunosum moleculare). GluA2–HT turnover exhibited a complex spatial pattern across layers (Fig. 4b). First, in stratum oriens and stratum radiatum, estimated lifetime increased with distance from the soma (from 3.5 to 7 days in stratum radiatum). Second, cell bodies showed very short GluA2–HT lifetimes. Third, the transition from stratum radiatum to stratum lacunosum moleculare was marked by a steep drop in lifetime (back to 3.5 days) and a lack of distance-dependent gradient from the soma. PSD-95–HT turnover showed a similar spatial pattern across CA1 layers (Fig. 4c) except for the fast turnover at the somatic layer. Fourth, at higher resolution, CA1 somata appeared to contain an intracellular pool of newly synthesized (<3 days old) GluA2 but not PSD-95 (Fig. 4d,e).

To explore the possibility of intracellular pools of GluA2, we separated GluA2–HT intracellular and surface pools using a modified pulse–chase paradigm, made possible because the HT was on the extracellular side of the receptor (Methods and Fig. 4f). In freshly cut hippocampal brain slices from GluA2–HT mice, we used a cell-impermeable pulse dye ligand, JF_549_i-HTL^59–61^, and thus labeled surface receptors only (JF_549_i; Fig. 4f). After 10 min, we used a chase dye ligand that is cell permeable and, therefore, also binds intracellular GluA2–HT (JFX_673_; Fig. 4f). CA1 somata were dominated by intracellular GluA2–HT (Fig. 4f). Additionally, both proximal and distal dendrites contained intracellular pools of GluA2–HT (Fig. 4f). These results revealed an intracellular (reserve) pool of AMPA receptors^62–66^ throughout CA1 pyramidal neurons (similar reserve pools were observed in the neocortex; Supplementary Movie 2).

Together, these results demonstrate the ability of DELTA to identify rich features of subcellular localization and local turnover rates of synaptic proteins.

## Discussion

We developed a robust pulse–chase method, DELTA, to measure turnover for endogenous proteins tagged with HT at high temporal and spatial precision. We identified the most suitable fluorescent ligands from existing JF dyes and identified JF_552_-HTL, JF_669_-HTL and its improved variant JFX_673_-HTL^33–35,39^ as the best for this method. Using DELTA, we uncovered learning-related changes in synaptic proteins in a subset of brain regions and cellular compartments, providing a brain-wide view of synaptic plasticity with high resolution.

We measured the lifetime of the major excitatory synaptic scaffold protein PSD-95 over a range of spatial scales from brain regions to single synapses. PSD-95–HT lifetimes ranged from 11 to 14 days, depending on the brain region (Fig. 2f,g). These values are comparable to estimates from metabolic labeling and MS (Extended Data Fig. 9h,i), but considerably longer than previous measurements using HT fusions and pulse-only dye ligand administration^29^. We found that PSD-95–HT dynamics were altered by enriched experience, with all cortical layers and CA1 as major loci of plasticity. However, an increase in synthesis was not fully counterbalanced by degradation, resulting in increased PSD-95–HT levels. By combining the pulse and chase signals, DELTA detects changes in total protein across conditions. However, additional measurements are required to fully deconvolve time-varying PSD-95–HT synthesis and degradation rates.

We generated knock-in mice with HT fused to the extracellular side of GluA2. We found that learning-induced turnover of GluA2 was most pronounced in the hippocampus, encompassing the entire CA1 hippocampal region, involving many cells. In contrast, an enriched environment caused more widespread changes in GluA2 dynamics throughout the brain. Previous studies have reported small ensembles of memory-related neurons labeled using immediate-early gene activation^67,68^. Our results suggest that these small ensembles may represent a subset of highly plastic cells during learning, both locally and brain wide.

We also found evidence for subcellular control of synaptic protein turnover for both GluA2–HT and PSD-95–HT, as turnover in the CA1 subregion of the hippocampus was regulated at the level of dendritic compartments (Fig. 4a–c). This dendritic specificity was surprisingly similar for GluA2 and PSD-95, suggesting coordination of turnover across proteins in a complex^69^. The main difference between GluA2 and PSD-95 was seen at the soma, where very little PSD-95 protein was observed (Fig. 4c,e). These data imply that PSD-95 either is rapidly transported into the dendrites after synthesis in the soma and/or is translated in dendrites^70^.

DELTA complements MS-based methods for measuring protein lifetime. MS measures thousands of proteins in parallel but provides only low spatial resolution and sensitivity. For example, EE has been shown to modulate turnover of approximately 30 synaptic proteins^10^ including a 15% increase in PSD-95 (ref. ^71^), consistent with our findings. DELTA only measures the lifetime of one HT fusion protein at a time but does so on scales ranging from millimeters to less than 100 nanometers by leveraging modern histology and microscopy methods. Localizing plasticity-related brain regions using DELTA, followed by proteome-wide measurements using MS in these brain regions, could provide comprehensive views of protein turnover in key plasticity loci. DELTA requires genetic manipulation, which itself may influence the target protein’s lifetime, and dye ligand saturation tests for each new HT fusion. However, DELTA is easily combined with other imaging-based molecular methods, such as fluorescence in situ hybridization^72^ or IF, to provide additional molecular context (Fig. 1d and Extended Data Fig. 8d–f). DELTA also has several advantages over pulse-only methods, such as higher signal-to-noise ratio, lower variability and the ability to assess changes in total protein (Extended Data Figs. 2f–i and 9e–j and Supplementary Text).

The ability to perform pulse–chase experiments to study protein turnover in vivo will enable experiments that could lead to new insights into the molecular mechanisms of synaptic plasticity and learning. Beyond measuring lifetime for other proteins and in other subcellular compartments, DELTA can be combined with the expression of indicators or actuators of neural activity to examine the relationship between neural activity and protein turnover. This could also be done in both healthy mice and animal models of brain disorders. Furthermore, DELTA is not limited to studies of the brain (Extended Data Fig. 7). Overall, DELTA could help identify organ-wide changes involved in adaptive and maladaptive processes in an unbiased, high-throughput and high-resolution manner.

## Methods

### Materials

JF-HTL dyes are available at HHMI’s Open Chemistry^36^ initiative (https://dyes.janelia.org/) or can be purchased from Promega. Purified HT protein (G4491) was from Promega. Pluronic F-127 (P3000MP), anhydrous dimethylsulfoxide (DMSO; D12345), *N*,*N*,*N*′,*N*′-tetramethylethylenediamine (TEMED, 15524010), ammonium persulfate (APS, AAJ7632209) and acryloyl-X succinimidyl ester (AcX-SE, A20770) were from Thermo Fisher Scientific. Acrylamide (1610140) and bis-acrylamide (1610142) were from Bio-Rad. To prepare sodium acrylate for ExM, acrylic acid (TCI A0141) was neutralized with sodium hydroxide^74^. 4-Hydroxy-TEMPO (176141) was from Sigma.

### Animals

All procedures were carried out according to protocols approved by the Janelia Institutional Animal Care and Use Committee (IACUC), under protocol number 23-249. WT mice (C57BL/6J RRID: IMSR_JAX:000664; both male and female) were housed in a 12-h–12-h reverse light–dark cycle with ad libitum access to food and water until they recovered from a headbar surgery. MeCP2–HT mice^75^, PSD-95–HT mice^50^ and GluA2–HT mice (this work) of either sex were used. No comparisons were made between males and females for MeCP2–HT knock-in mice as it is an X-linked gene. A list of all animals used in this study is available in Supplementary Table 4.

### Dye ligand clearance

Cranial windows were made over the anterior lateral motor cortex or primary visual cortex (centered on −2.5 mm lateral, +0.5 mm anterior from lambda) and a headbar was attached posterior to the window^31^. Mice were anesthetized with 1% isoflurane and kept at 37 °C on a heating pad. The optical setup^76^ is based on a custom wide-field fluorescence microscope (Thorlabs) and an sCMOS camera (Hamamatsu Orca Flash 4.0 v3). Details of illumination, objectives and filters are presented in Supplementary Table 1. A baseline image was acquired before dye ligand injection. Both the baseline and subsequent time points were acquired as movies of 40–50 frames at 5 Hz. This was done to reduce the chances of saturating pixels on the camera at the peak on the injection while still being above electrical noise under baseline conditions. After the estimation of the baseline, the animal was removed from head fixation and injected with dye. After dye ligand injection, it was quickly returned to head fixation; the field of view was compared to a baseline image under room lights and then imaged for up to 4 h in intervals of 2 min to 20 min to cover the fast decay part of the dye ligand clearance. The animal was then recovered and reimaged for up to 24 h at 4–12-h intervals.

The images were analyzed using a custom MATLAB script. Briefly, each movie was averaged, and a manual region of interest was defined (Extended Data Fig. 1b). The pixel-averaged mean was fitted to a double exponential: *F*$=a\times {e}^{-1/{\tau }_{1}}+b{\boldsymbol{}{\times}}{e}^{-1/{\tau }_{2}}{\boldsymbol{+}}c$. Population averages were computed by binning all 11 experiments together. In cases where the injection phase was captured, an offset was used to start the fitting from the highest time point.

### Carotid artery dye ligand infusion

To measure the infusion kinetics of JF dyes, we needed a way to control the rate of infusion while imaging dye ligand accumulation in the brain through a cranial window (Extended Data Fig. 5). We used two C57BL/6J mice with carotid artery vascular catheterization (from Charles Rivers surgical service). They were flushed with saline and 50% sucrose lock solution every 7 days. A cranial window surgery was performed 7 days after catheterization as described above. Seven days later, a pump with JF dye ligand was connected to the carotid artery line. The initial pump rate was 20 µl min^−1^ followed by 40 µl min^−1^ and 80 µl min^−1^. The imaging conditions were the same as for the dye ligand clearance experiments (0.2 s exposure) but imaged continuously for each injection speed. Data were time averaged at 1-s intervals (five time points) and a manual region of interest was drawn. The time-averaged fluorescence values were trimmed to the times the pump was running and fit with a linear fit (including intercept). The values reported are the slopes of the fit.

### Modeling dye ligand clearance effects on saturation

Given our results on dye ligand infusion (Supplementary Text and Extended Data Fig. 5e), we tested the hypothesis that given a fixed amount of dye ligand to inject, faster injections would lead to more dye ligand captured in the brain. We used a model where a single position in space (*x* position 1) is a blood vessel from which dye ligand can enter the brain and diffuse in one dimension simulated using a time-march algorithm (finite-difference method). To compare the effect of injection speed alone, in each simulation, we used the same total amount of dye ligand injected, and the same amount of dye ligand was cleared from the vessel (1 a.u. per d*t*, where d*t* is one time step). Simulations had different injection rates (2–10 a.u. per d*t*) and the width of the dye ligand distribution was measured as the width at 90% of the known saturation value.

### Modeling protein turnover measurements with DELTA

Pulse–chase experiments were modeled using the SimBiology package in MATLAB. Lifetimes of the target HT protein synthesis were simulated. Each new protein could either degrade (at the same rate of synthesis modeled) or attach to an HTL dye molecule (pulse or chase, dye ligand collectively). The dye ligand binding kinetics were set to be instantaneous, as they are several orders of magnitude higher than those for synthesis/degradation. Binding kinetics were identical for both the pulse and chase. The degradation rates of the HT protein with dye ligand complexes were the same as for the protein alone.

The dye ligand injection kinetics were neglected (that is, assume instantaneous injection). Dye ligand clearance kinetics were modeled with two compartments (cytosol and lipids) with clearance from the cytosol compartment. Multiple compartments were required to account for the multi-exponential decay measured in vivo. The cytosol compartment was set to have a volume of 1 ml; the equilibrium constant between the compartments, the volume of the lipids compartment and the degradation time constant were fitted using the JF dye clearance data (Extended Data Figs. 1 and 2). This fitting was done in the absence of HT protein.

Our estimates of protein turnover assume constant protein levels across manipulations. This assumption likely breaks down for some proteins and conditions, which will then require modifying the calculations to estimate synthesis and degradation independently. Indeed, we find that PSD-95–HT levels increase after EE, while Pulse levels are unchanged, implying that the rate of protein degradation is unchanged. We estimated the effect of changing synthesis rates on mean lifetime as follows:

Let *P* = (PSD-95–HT), then under baseline conditions:

$$\frac{{{\rm d}P}}{{{\rm d}t}}=r-{aP}$$

Where *r* is the synthesis rate and *a* is the degradation rate. In steady state, $\frac{{{\rm d}P}}{{{\rm d}t}}=0$. After EE:

$$\frac{{{\rm d}P}}{{{\rm d}t}}={r}^{* }\left(t\right)-{aP}$$

Where r* (*t*) > *r* is the new higher synthesis rate, which could vary arbitrarily over time. Using the sum of the pulse and chase in DELTA gives us a single point in time to estimate the deviation from the assumption of steady-state conditions. If we additionally assume that r* (*t*) = r* is constant, we can estimate the protein level in closed form:

$$P\left(t\right)=\frac{{r}^{* }}{a}+\left({P}_{0}-\frac{{r}^{* }}{a}\right){e}^{-{at}}$$

Where *P*_0_ is the protein concentration at *t* = 0, ${P}_{\infty }=\frac{{r}^{* }}{a}$ the concentration at *t* → *∞*, and *A*(*t*) = *e*^−*at*^ we get to:

$$P(t)={P}_{\infty }+\left({P}_{0}-{P}_{\infty }\right)A\left(t\right)$$

We model the total protein concentration *P*(*t*) as the sum of two populations:

$$P\left(t\right)=O\left(t\right)+N\left(t\right)$$

The original protein population (*O*) is present at *t* = 0 and degrades over time as $O\left(t\right)={P}_{0}{A}(t)$, with mean age $\frac{1}{a}+t$.

The accumulated age is ${M}_{O}\left(t\right)={P}_{0}A\left(t\right)\left(\frac{1}{a}+t\right)$.

The new protein population (*N*) is synthesized after *t* = 0, *N* (*t*) = *P*(*t*) − *O*(*t*) = *P*_*∞*_ (1 − *A*(*t*)), with accumulated age:

$${M}_{N}\left(t\right)={r}^{* }{\int }_{0}^{{t}}{sA}\left(s\right){ds}={r}^{* }\left(-\frac{{tA}\left(t\right)}{a}+\frac{1-A\left(t\right)}{{a}^{2}}\right)$$

The mean age (${\tau }_{{mean}}^{* })$) is the accumulated age of proteins divided by total protein concentration:

$$\begin{array}{l}{\tau }_{{mean}}^{* }=\,\displaystyle\frac{{M}_{O}\left(t\right)+{M}_{N}\left(t\right)}{P\left(t\right)}\\\qquad\;\;=\,\displaystyle\frac{\left({P}_{0}-{P}_{\infty }\right)A\left(t\right)\left(\displaystyle\frac{1}{a}+t\right)+\displaystyle\frac{{P}_{\infty }}{a}}{{P}_{\infty }+\left({P}_{0}-{P}_{\infty }\right)A\left(t\right)}=\displaystyle\frac{1}{a}+\frac{{tA}\left(t\right)\left({P}_{0}-{P}_{\infty }\right)}{P\left(t\right)}\end{array}$$

For our measurement time point (*t* = 14 days; Fig. 2a), we can then estimate a correction for the lifetime estimate under the assumption that ${r}^{* }$ is constant. The error (Extended Data Fig. 2i) is computed as the percentage deviation from the steady-state mean lifetime**:**

$${\tau }_{\rm{mean}}=\frac{1}{a}=\frac{\Delta t}{\log \left(1/{\rm{fraction}}\; {\rm{pulse}}\right)}$$

Where $\Delta t$ is the pulse–chase interval and $\rm{Fraction}\; \rm{pulse}=\frac{\rm{pulse}}{\rm{pulse}+\rm{chase}}$. In addition, we computed corrections for non-constant ramping-up and ramping-down r* (*t*), which produced similar corrections. In general, in cases where overall protein levels are not constant across conditions, robust estimates of the synthesis and degradation rates will benefit from measurement of protein levels at multiple time points following the manipulation.

### Dye ligand solubility

JF dye ligands were prepared in the following manner. (1) Captisol formulation was made with 300 mg Captisol (β-cyclodextrin sulfobutyl ethers, sodium salts; NC-04A-180186, Cydex Pharmaceuticals) dissolved in 1 ml of water (Molecular Biology Grade Water, Corning) to make a 30% solution, and 100 µl of this solution was added to 100 nmol of dry JF dye. (2) A Captisol + Pluronic formulation was made by mixing a 30% Captisol solution with Pluronic F-127 (P3000MP, Invitrogen) in an 80:20 ratio, and 100 µl of the prepared solution was added to 100 nmol of dry JF dye. (3) The DMSO + Pluronic formulation was made with DMSO (D2650, Sigma-Aldrich), Pluronic F-127 and saline (0.9% sodium chloride) mixed in a 20:20:60 ratio. In total, 100 µl from the prepared solution was added to 100 nmol of dry JF dye. (4) The DMSO formulation was made by adding 100 µl of DMSO to 100 nmol of dry JF dye ligand to bring it to a final concentration of 1 mM.

The prepared JF dye ligand formulations were briefly vortexed followed by bath sonication (Branson 1200 model) for 5 min. The dye ligand solutions were placed on the agitator for 72 h to ensure solubilization. Absorbance measurements of dye ligand solutions were performed using a spectrometer (Cary 100 UV-Vis, Agilent Technologies), and the final concentration was determined from known extinction coefficients of JF dyes (Extended Data Fig. 6e–h)^33–35,39^.

### Evaluation of JF-HT ligands in vivo

To evaluate the ability of different JF-HTL dyes to saturate HT proteins in the brain, we expressed HT-EGFP (Extended Data Figs. 3 and 4). Virus delivery for sparse expression and dye ligand delivery were achieved using retro-orbital injections^77^. Dye ligand preparation^78^, dye ligand injections^79^ and histological preparations^80^ have been described. A virus to express HT-EGFP was prepared using a PHP.eB AAV capsid^81^ with a synapsin promoter. In total, 100 μl of virus (titer of 4 × 10^11^ genome copies per ml) was injected retro-orbitally. An mKate-HT was used to avoid cross-talk between the GFP and the JF_525_-HTL dye. Retro-orbital dye ligand injections (pulse) were performed 3–5 weeks after the viral injection. Dyes were prepared by dissolving 100 nmol of dye ligand in 20 µl DMSO followed by 20 µl of 20% Pluronic F-127 in DMSO and 60 µl of PBS, except when otherwise stated (Supplementary Table 2). Twenty-four hours after dye ligand injection, animals were perfused with 50 ml of 4% paraformaldehyde (PFA) in 0.1 M sodium phosphate, pH 7.4 (phosphate buffer) and 50 nmol of orthogonal dye. The brains were post-fixed in 4% PFA in 0.1 M phosphate buffer overnight at 4 °C and washed three times in PBS for 15 min. Then, 100-µm coronal sections were cut and floated in 24-well plates followed by 4 h of DAPI staining (0.6 µM in PBS) and washed three times for 15 min in PBS. For animals with visible fluorescence from in vivo injection, every fourth slice was mounted for imaging; for animals where no fluorescence was observed, every 24th slice was mounted and imaged. See Supplementary Table 2 for details of the animals in this section.

### IF

For MeCP2–HT mice, we performed IF staining to distinguish cell types in histological sections (Fig. 1d and Extended Data Fig. 8d–f). Brains were sectioned coronally, and sections were blocked in PBS with 2% BSA and 0.1% Triton X-100 for 1 h at room temperature. Primary antibodies (Rabbit Anti-NeuN, RRID: AB_10807945, Millipore, ABN78; Rabbit Anti-Iba1, RRID: AB_2636859, Abcam, ab178846; Rabbit Anti-SOX10, RRID: AB_2721184, Abcam, ab180862) were applied at a concentration of 1:250 in the same buffer overnight at 4 °C. The sections were washed three times for 15 min. The secondary antibody was a Goat anti-Rabbit AF488 (RRID: AB_143165) used at a 1:500 dilution overnight at 4 °C. After three more 15-min washes, the slices were stained with DAPI as described^80^ and mounted. For PSD-95 IF (Extended Data Fig. 9c), we used a validated anti-PSD-95 antibody at a dilution of 1:250 (NeuroMAB, RRID: AB_10807979) with a Goat anti-mouse CF633 secondary at a dilution of 1:500 (RRID: AB_10854245). For validation of GluA2–HT (Extended Data Fig. 10c), we used another monoclonal antibody from NeuroMAB at a dilution of 1:250 (RRID: AB_2232661) with the same secondary at a 1:500 dilution.

### ExM

Coronal sections (Figs. 2k and 4d,e and Extended Data Figs. 9c and 10c) were anchored with AcX (0.033 mg ml^−1^; 1:300 dilution from 10 mg ml^−1^ stock in DMSO) in PBS for 1 h. Sections were then transferred to a gelation solution (2.7 M acrylamide, 0.2 M sodium acrylate, 200 µg ml^−1^ bis, PBS (1×), 2 mg ml^−1^ APS, 2 mg ml^−1^ TEMED and 20 µg ml^−1^ 4-hydroxy-TEMPO) and incubated on ice, shaking, for 30 min before being mounted in gelation chambers and moved to an incubator at 37 °C for 1 h. Excess gel was removed, and the tissue was recovered in pure water. With three 1-h washes in pure water, the slices expanded approximately twofold without disruption or cracks. These sections were moved to a six-well glass-bottom plate for microscopy (Cellvis, P06-1.5H-N). To flatten and immobilize the sections, four dabs of silicon grease were applied around each section and a 22 × 22-mm square number 2 coverslip (Corning, 2855-22) was pressed from above. If needed, 0.2 mg ml^−1^ poly-l-lysine (Sigma, P1524-25MG) with Photo-Flo 200 (1:500 dilution from stock; 74257, Electron Microscopy Sciences) was applied to the bottom of the well before the sections were placed to better immobilize the gels.

### Brain-wide imaging

Imaging of entire sections at high resolution was performed on a confocal slide scanner consisting of a TissueFAXS 200 slide feeder (TissueGnostics, Germany) and a SpectraX light engine (Lumencor). Illumination light (wavelength, power, (excitation filters center/width): (1) 395 nm, 400 mW (395 nm/25 nm); (2) 475 nm, 480 mW (475 nm/34 nm); (3) 400 mM (585 nm/35 nm); (4) 619 nm, 629 mW (635 nm/22 nm) was delivered by a lightguide to a Crest X-Light V2 confocal spinning disk microscope (Crestoptics; 60-μm pinhole spinning disk) with the following dichroics (Chroma Technology, T425lpxr, T495lpxt, T600lpxr, T660lpxr) and emission filters (ET460 nm/50 nm, ET525 nm/50 nm, ET625 nm/30 nm, ET700 nm/75 nm). The emission light was collected with Zeiss objectives EC Plan-Neofluar ×10/0.3 M27 for MeCP2 and PSD-95 animals (Fig. 2c–k and Extended Data Figs. 6 and 7) and a Plan-Apochromat ×20/0.8 M27 objective for virally injected animals (Extended Data Figs. 3 and 4). Detection was with a Zyla 5.5 sCMOS camera (Andor). Image acquisition involved semiautomated tissue detection using multiple autofocusing points per section (5 × 5 and 3 × 3 grids for objective acquisitions of ×20 and ×10 accordingly). For virally transfected animals, three *z*-planes were imaged with a 7-µm spacing and projected in *z*. For MeCP2 and PSD-95 animals, a single plane was imaged.

### High-NA and super-resolution imaging

To segment individual nuclei of MeCP2–HT-expressing cells (Fig. 1d–f and Extended Data Fig. 8) or single synapses expressing PSD-95–HT (Fig. 2i), higher-resolution imaging was needed. For MeCP2 imaging, we used a Zeiss LSM 880 with an EC Plan-Neofluor ×40/1.3-NA oil objective and a voxel size of 0.25 × 0.25 × 0.5 μm. We acquired 21 *z*-planes in three tracks (a track can have more than one detection channel for multiplexing) and five channels (detection (excitation) wavelengths were as follows: track 1 DAPI: 410–489 nm (405 nm), JF_612_: 624–668 nm (594 nm); track 2 IF: 493–551 nm (488 nm), JF_669_: 680–758 nm (633 nm); track 3 JF_552_: 553–588 nm (561 nm). For PSD-95 imaging, we used a Zeiss LSM 980 with Airyscan and a C-Apochromat ×40/1.2 water-dipping objective. The voxel size was 57 × 57 × 230 nm. We acquired 23 *z*-sections with two channels, 561-nm illumination for JF_552_ and 633-nm illumination for JFX_673_. A full z-stack was acquired for the far-red channel followed by the red channel.

### Image analysis

#### Janelia Fluor dye ligand screening

We used ‘fraction pulse = pulse/(pulse + chase)’ as a measure of saturation, where (pulse + chase) is a measure of total protein. Fraction pulse was calculated after conversion to micromolar units of dye, where adding the pulse and the chase are a valid operation (same units). Fraction pulse values closer to 1 indicate saturation. Doing a pixel-wise analysis would not be sufficient, as there are background signals that would change depending on the imaged channel and brain region. Here, unlike knock-in animals (GFP-HT expression with the PHP.eb virus), there is variability in the expression pattern across animals that could also affect the background (neuropil) in which the cells reside (Extended Data Fig. 3).

We performed analysis on detected cells with local background subtraction. To detect cells, we imported downsampled (2×) images with three channels (GFP/pulse/chase) into Ilastik’s pixel-classification workflow^82^. The workflow was manually trained to segment cell bodies, dendrites, neuropil signal and various imaging and tissue artifacts. The pixel probability maps for cells, combined with the raw data, were then imported to a second object-classification workflow in Ilastik. This workflow was used to classify each mask as a GFP-HT-expressing cell or not. A MATLAB (MathWorks) script was used to calculate the value of each mask in the three channels and to calculate a local background. The local background estimation excluded pixels that belonged to other non-neuropil-trained categories (for example, other cells, dendrites) from the first Ilastik workflow (Extended Data Fig. 3c). For each mask, the local background was subtracted, and the fluorescence value was converted to micromolar dye ligand concentration using an independent calibration obtained under the same imaging conditions (Extended Data Fig. 4a).

#### MeCP2–HT knock-in mice

Nuclei were segmented and categorized into cell types using IF (Fig. 1c–e and Extended Data Fig. 8d–f). First, the five imaged channels (DAPI, pulse, chase 1, chase 2, IF) were each normalized (0 to 1) using the top and bottom 0.3% of the pixel intensity histogram. Second, the mean of all three imaged JF dyes was used to make a three-channel image (DAPI, mean of all JF dyes, IF). This three-channel image was used in a pixel-classification workflow using Ilastik (https://www.ilastik.org/). We used training data from all three types of IF (NeuN, Iba1 and SOX10). The resulting nuclei probability maps, together with the three-channel images, were used by three independent object-classification workflows in Ilastik, one for each IF type. The output was a set of masks for IF-positive nuclei for each cell type. We calculated three fraction pulses from these dual chase experiments for each segmented nuclei: (1) pulse/pulse + chase 1; (2) pulse + chase 1/pulse + chase 1 + chase 2; (3) chase 1/chase 1 + chase 2. These were done after conversion to micromolar dye ligand units using a calibration curve for each dye ligand making the addition of different dyes a valid operation. We then pooled all 15 measurement (5 animals × 3 fraction pulses) and used bootstrapping to fit a lifetime with the assumption of a single exponential decay.

#### PSD-95–HT mice

In contrast to MeCP2, the diffuse PSD-95 expression did not permit segmentation of individual neurons or synapses with standard fluorescence microscopy. We instead performed a pixel-based analysis after 2× down sampling (resulting pixel size, 2.7 × 2.7 µm; Fig. 2b–k). As with MeCP2, we converted our images to three-channel images (DAPI/pulse/chase). A pixel-classification workflow (Ilastik) was trained to exclude ventricles and artifacts. Each pixel was converted to a lifetime estimate with the assumption of a single exponential decay after conversion to micromolar dye ligand concentration using calibration curves imaged under the same conditions as was explained above.

#### GluA2–HT mice

GluA2 analysis was similar to PSD-95, with slight modifications. A different workflow was trained (Ilastik) to segment regions with signal. Additionally, four animals without HT (negative controls) were imaged, registered to the Allen Brain Reference Atlas^53^ (CCFv3) and their pulse and chase signals were quantified. This negative control was used to define a brain-region-specific threshold as two standard deviations above the mean of the negative control average. The threshold was used to exclude brain regions where GluA2–HT signal was not substantially higher than autofluorescence. This was done for both the pulse and the chase channels, causing rejection of 140/1,327 possible regions (CCFv3). As CA1 lamina (Extended Data Fig. 10k; stratum oriens, stratum radiatum, stratum lacunosum moleculare and the cell body layer) were not present in our reference atlas (CCFv3), we manually segmented them using the Ground Truth Labeler GUI in MATLAB.

#### Single-synapse analysis after ExM

After acquisition using the Airyscan detector array, we used Zen Blue software (Zeiss) to process the images (Airyscan processing function in three dimensions with automated deconvolution strength). The Airyscan-processed images were registered across channels as they were acquired sequentially. The resulting two-channel image was normalized and used as input to an Ilastik pixel-classification pipeline trained to separate synapses from the background. The resulting probability images with the normalized data were used in a second Ilastik object segmentation pipeline (Fig. 2l). A local background was subtracted, fluorescence values were converted to micromolar units of dye, and lifetimes were estimated.

### Alignment to the Allen Brain Reference Atlas CCFv3

MeCP2, PSD-95 and GluA2 lifetime measurements were registered to the Allen Brain Reference Atlas^53^ (CCFv3) using a two-step procedure. First, a downsampled 24-bit RGB image of each section was loaded with QuickNII^83^ and aligned with the 25-µm voxel resolution version. This accounted for the cutting angle, a global scaling factor in the dorsoventral and mediolateral axes, and the anteroposterior location of each section. QuickNII output was used for a manual non-rigid alignment with VisuAlign^84^. The main markers for registration were the edges of the section, the ventricles and the fiber tracks. The VisuAlign output was another RGB image in which each color was assigned to an Allen CCFv3 ID. These images were loaded and interpolated to the original size of each section, allowing the assignment of each MeCP2 nucleus and PSD-95/GluA2 pixel to a CCF ID. As the PSD-95 and GluA2 analysis was pixel based, we excluded pixels belonging to the root or any part of the CCFv3 tree under fiber tracks or the ventricular system.

### Statistics and reproducibility

To compare brain regions across animals assigned to different experimental conditions, we used an LME model. Given that data were acquired from coronal slices, where the same brain regions may appear in varying numbers of slices across different animals, we used pixels as our base unit of measurement. Each pixel was represented as a row in a table with columns corresponding to the animal ID, brain region and mean lifetime.

We fitted an LME model using this table, applying median filtering to cap the maximum number of repeated measurements at 1,000 per condition (where a condition is defined as the same brain region within the same animal). The LME model included one fixed effect (group assignment: control, EE, baseline, new rule), one random effect (animal ID) and an estimate of residual error. We assumed a normal of the residuals according to the central limit theorem.

This approach allowed us to compute the mean and standard error of the mean for each condition in the fixed effects (for example, layer 1, layer 2/3), as well as the mean and standard deviation for the residual error and across conditions in the random effects (for example, individual animals).

No statistical methods were used to predetermine sample sizes but our sample sizes are similar to those reported in previous publications^10,29,85–87^. No data were excluded from the analyses. Animals were randomly assigned to experimental groups (that is, control versus EE). The investigators were not blinded to allocation during experiments and outcome assessment.

### MS-based measurement of turnover

#### C13 lysine heavy isotope feed labeling in mice and MS sample preparation

We followed a previously published protocol for isotope labeling for MS-based turnover measurements^88^. Either WT or homozygote PSD-95–HT mice cortices were rinsed and dissected in solution A (5 mM HEPES pH 7.4, 1 mM MgCl_2_, 0.5 mM CaCl_2_, 1 mM dithiothreitol, 0.32 M sucrose and protease and phosphatase inhibitor set (Thermo Fisher Scientific, 78443_3670527377) on ice. Then, the tissues were homogenized with an electronic homogenizer (Glas-Col, 099C-K54). Homogenates were spun down at 1,400*g* for 10 min (4 °C). The supernatant was set aside. We then resuspended the pellets in 20 ml solution A. The diluted homogenates were centrifuged at 710*g* for 10 min (4 °C). The pellet is P1. We combined and mixed the supernatant and the saved supernatant as S1. S1 was centrifuged at 13,800*g* for 10 min (4 °C). The supernatant is S2. Then, we resuspended the pellets (P2) in solution B (6 mM Tris pH 8.1, 0.32 M sucrose, 1 mM EDTA, 1 mM EGTA, 1 mM dithiothreitol with protease and phosphatase inhibitors).

Proteins were extracted from P2 using a chloroform–methanol precipitation method. Protein pellets were resuspended in 8 M urea (Thermo Fisher Scientific, 29700) prepared in 100 mM ammonium bicarbonate solution (Fluka, 09830). The samples were reduced with 5 mM Tris(2-carboxyethyl)phosphine (TCEP, Sigma-Aldrich, C4706; vortexed for 1 h at room temperature), alkylated in the dark with 10 mM iodoacetamide (Sigma-Aldrich, I1149; 20 min at room temperature), diluted with 100 mM ABC and quenched with 25 mM TCEP. Samples were diluted with 100 mM ammonium bicarbonate solution, and digested with Trypsin (1:50 dilution; Promega, V5280) for overnight incubation at 37 °C with intensive agitation. The next day, the reaction was quenched by adding 1% trifluoroacetic acid (Fisher Scientific, O4902–100). Samples were desalted using Peptide Desalting Spin Columns (Thermo Fisher Scientific, 89882). All samples were vacuum centrifuged to dry. We further used a high-pH reverse-phase peptide fractionation kit (Thermo Fisher Scientific, 84868) to get eight fractions (5.0%, 7.5%, 10.0%, 12.5%, 15.0%, 17.5%, 20.0% and 50.0% of acetonitrile in 0.1% triethylamine solution). All fractions were vacuum centrifuged to dry. The high-pH peptide fractions were directly loaded into the autosampler for MS analysis without further desalting.

#### Tandem MS

Three micrograms of each sample were loaded using an autosampler with a Thermo Vanquish Neo UHPLC system onto a PepMap Neo Trap Cartridge (Thermo Fisher Scientific, 174500; diameter, 300 µm; length, 5 mm; particle size, 5 µm; pore size, 100 Å; stationary phase, C18) coupled to a nanoViper analytical column (Thermo Fisher Scientific, 164570; diameter, 0.075 mm; length, 500 mm; particle size, 3 µm; pore size, 100 Å; stationary phase, C18) with a stainless-steel emitter tip assembled on the Nanospray Flex Ion Source with a spray voltage of 2,000 V. An Orbitrap Ascend (Thermo Fisher Scientific) was used to acquire all the MS spectral data. Buffer A contained 99.9% H_2_O and 0.1% formic acid, and buffer B contained 80.0% acetonitrile, 19.9% H_2_O with 0.1% formic acid. For each fraction, the chromatographic run was for 2 h in total with the following profile: 0–8% for 6 min, 8% for 64 min, 24% for 20 min, 36% for 10 min, 55% for 10 min, 95% for 10 min and again 95% for 6 min.

We used the Orbitrap HCD-MS2 method for these experiments. The following parameters were used: ion transfer tube temp = 275 °C; Easy-IC internal mass calibration, default charge state = 2; and cycle time = 3 s. Detector type was set to Orbitrap, with a resolution of 60,000, with wide quad isolation; mass range = normal; scan range = 375–1,500 *m/z*; maximum injection time mode = auto; automatic gain control target = Standard; microscans = 1; S-lens RF level = 60; without source fragmentation; and data type = profile. MIPS was set as ‘on’, included charge states = 2–7 (reject unassigned). Dynamic exclusion enabled with *n* = 1 for 60-s exclusion duration at 10 ppm for high and low with exclude isotopes; isolation mode = quadrupole; isolation window = 1.6; isolation Offset = Off; active type = HCD; collision energy mode = Fixed; HCD collision energy type = normalized; HCD collision energy = 25%; detector type = Orbitrap; Orbitrap resolution = 15,000; mass range = normal; scan range mode = auto; maximum injection time mode = auto; automatic gain control target target = standard; microscans = 1; and data type = centroid.

#### MS data analysis and quantification

Protein identification/quantification and analysis were performed with Integrated Proteomics Pipeline - IP2 (Bruker; http://www.integratedproteomics.com/) using ProLuCID^89,90^, DTASelect2 (refs. ^91,92^) and Census and Quantitative Analysis. Spectrum raw files were extracted into MS1 and MS2 files using RawConverter (http://fields.scripps.edu/downloads.php/). The tandem mass spectra (raw files from the same sample were searched together) were searched against UniProt mouse (downloaded on 29 July 2023) protein databases^93^ and matched to sequences using the ProLuCID/SEQUEST algorithm (ProLuCID version 3.1) with a 50-ppm peptide mass tolerance for precursor ions and 600 ppm for fragment ions. The search space included all fully and half-tryptic peptide candidates within the mass tolerance window with no-miscleavage constraint, assembled and filtered with DTASelect2 through IP2. To estimate protein probabilities and false discovery rates accurately, we used a target/decoy database containing the reversed sequences of all the proteins appended to the target database^93^. Each protein identified was required to have a minimum of one peptide with a minimal length of six amino acid residues. After the peptide/spectrum matches were filtered, we estimated that the protein false discovery rates were ≤1% for each sample analysis. Resulting protein lists include subset proteins to allow for consideration of all possible protein isoforms implicated by at least three given peptides identified from the complex protein mixtures. Then, we used Census and Quantitative Analysis in IP2 for protein quantification. Static modification was set to 57.02146 C for carbamidomethylation. Differential modification was set to 42.0106 for acetylation at N terminals. Mass shift for heavy lysine was set as 6.0201. Quantification was performed by the built-in module in IP2. For quantification, only peptides containing the amino acid lysine (K) were selected. For the estimation of protein half-life, half-life values were analyzed as previously described^10,88^. Half-life values were divided by $\mathrm{ln}(2)$ to calculate mean lifetime (*τ*).

### GluA2–HT knock-in mouse generation and validation

The GluA2 Halo knock-in line was generated by CRISPR–cas9 pronuclear microinjection. The Halo gene was inserted after the signal peptide of the GluA2 gene and flanked by two linkers, GGGGSGGGS at the 5′ end and GGGGSGGGSGGGGSGGGS at the 3′ end. The homologous arms are 332 bp and 655 bp, respectively. The knock-in DNA was co-injected with a gRNA (5′-TCTTCTAACAGCATACAGATAGG-3′) and Cas9 protein (Fisher Scientific, A36498) into B6D2F1/J mouse one-cell embryos. F2’s were used in this study from both sexes. A founder with germline transmission is being backcrossed to a C57BL/6J background and will be donated to Jackson Laboratories for dissemination.

For brain protein-level measurements (Extended Data Fig. 10b), hemibrains were freshly dissected and homogenized in 1 ml of ice-cold homogenization solution (320 mM sucrose, 10 mM HEPES pH 7.4, 1 mM EDTA, 5 mM Na pyrophosphate, 1 mM Na_3_VO_4_, 200 nM okadaic acid, protease inhibitor cocktail (Roche)). The resulting homogenate was centrifuged at 800*g* for 10 min at 4 °C to obtain the P1 (nuclear, pellet) and S1 (supernatant) fractions. We then centrifuged the S1 fraction at 17,000*g* for 20 min at 4 °C to obtain the P2 (membrane, pellet) and S2 (cytosol, supernatant) fractions. Mouse brain P2 protein fractions were separated by SDS–PAGE using 6% gels and proteins were transferred onto nitrocellulose membranes for western blot analysis. Western blots were imaged using the LI-COR Odyssey M imager. The following antibodies were used for detection: JH6773 (homemade) anti-GluA2 rabbit polyclonal antibody and anti-PSD-95 monoclonal antibody K28/74R (Addgene, 184184)^94^. JH6773 is a GluA2 N-terminal antibody generated using CDYDDSLVSKFIERWSTLE peptide (amino acids 264–281). For protein quantification, three WT and three homozygous GluA2–HT mouse brain samples were run in duplicate, quantified using LI-COR Image Studio software, and the averages for each duplicate were taken to measure expression levels using PSD-95 levels as a normalization signal.

### Brain slice preparation and electrophysiology

For the validation and investigation of GluA2–HT mice (Fig. 4f and Extended Data Fig. 10e–h), transverse hippocampal slices (300 µm) were prepared from 3–6-month-old WT or GluA2–HT knock-in mice. The brain was removed rapidly and mounted in a near-horizontal plane for slice preparation (vibrating tissue slicer Leica VT 1200S, Leica Microsystems). Slices were prepared in ice-cold sucrose-based solution containing: 75 mM sucrose, 75 mM NaCl, 2.5 mM KCl, 25 mM NaHCO_3_, 1.25 mM NaH_2_PO_4_, 7 mM MgCl_2_, 0.5 mM CaCl_2_ and 25 mM dextrose. Slices were then transferred to an incubation chamber filled with oxygenated artificial cerebrospinal fluid containing: 125 mM NaCl, 2.5 mM KCl, 25 mM NaHCO_3_, 1.25 mM NaH_2_PO_4_, 1 mM MgCl_2_, 2 mM CaCl_2_ and 25 mM dextrose. After half an hour of recovery at 35 °C, the slice chamber was maintained at room temperature. All experiments were performed in the presence of GABA_A_ and GABA_B_ receptor antagonists SR 95531 (4 μM) and CGP 52432 (1 μM).

Whole-cell current-clamp recordings were obtained with a Multiclamp 700B amplifier (Molecular Devices), using bridge balance and electrode-capacitance compensation. Patch-clamp electrodes were pulled from borosilicate glass (1.5 mm outer diameter) and filled with intracellular solution containing: 120 mM K-gluconate, 20 mM KCl, 10 mM Na_2_phosphocreatine, 10 mM HEPES, 2 mM MgATP, 0.3 mM Na_2_GTP, 0.1% biocytin. Electrode resistance in the bath was 3–5 MΩ and series resistance during the recordings was 15–30 MΩ. Electrophysiological traces were low-pass filtered with a cutoff frequency of 5 kHz and digitized at 20 kHz via a USB-6343 board (National Instruments) under the control of WaveSurfer software (https://wavesurfer.janelia.org/; Janelia Scientific Computing).

Excitatory postsynaptic potential amplitude was monitored while stimulating extracellularly at 0.05 Hz with concentric bipolar electrodes (FHC) with A365 stimulus isolator (World Precision Instruments). Stimulating electrodes were positioned in the distal stratum radiatum, closer to the stratum lacunosum moleculare than the stratum pyramidale, at least 200 μm away from the recorded cell body. Long-term potentiation was induced using a theta-burst protocol in which a single burst of excitatory postsynaptic potentials (five stimuli, 100 Hz) was delivered five times at 5 Hz. The theta stimulus was repeated three times, separated by 30 s. Membrane potential s.d. was estimated by using 2–5-min recordings at −65 mV without synaptic stimulation. Analysis of electrophysiology data was performed using Clampfit 11.3 software (Molecular Devices) and statistical tests were performed using Prism 10 (GraphPad Software).

Separate brain slices were used to localize the intracellular and extracellular pools of GluA2–HT receptors (Fig. 4f). A cell-impermeable version of JF_549_ (JF_549i_-HTL)^34^ was first dissolved in DMSO to a concentration of 1 mM. A working solution concentration of 1 μM was applied for 10 min in ACSF. After 10 min, a cell-permeable dye ligand was added (1 μM JFX_673_, from 1 mM stock dye ligand in DMSO). Following another 10 min, 3 × 5 min washes were done in ACSF before the slices were fixed for 30 min at room temperature with 4% PFA in 0.1 M sodium phosphate, pH 7.4. Slices were washed 3 × 15 min in 1× PBS and expanded twofold as described below. These short incubation periods meant the dyes did not penetrate the whole 300 μm of the slice. Images were taken from depths where saturation of the cell-impermeable dye ligand was observed (5–50 μm deep) as seen by the separation of extracellular staining and intracellular staining, whereas deeper in the tissue the cell-permeable dye ligand was seen in both compartments.

### Behavioral protocols for GluA2–HT

#### Headbar implant for behavior in virtual reality

We followed a previously described protocol^95^ with slight modifications. Mice were anesthetized with 1.5–2% isoflurane during the time of the procedure. We exposed the skull, using the following coordinates: 1.8 mm posterior relative to bregma and 2.0 mm lateral relative to the midline. Once we localized the anteroposterior and mediolateral coordinates, we drew a dot on the skull. We centered the headbar around this point and we glued the titanium headbar (custom design) with dental cement in the skull. After surgery, we allowed animals to recover for a minimum of 3 days before their water restriction protocol.

#### Water restriction and handling

After surgery, mice were single housed and underwent water restriction (typically 1–1.5 ml per day depending on their weight) for 3–5 days before initiating training. During water restriction, mice were gently handled and daily water was delivered with a syringe directly to the mouse’s mouth. In addition, we provided the mice with snacks; sunflower seeds, Cocoa Krispies or Froot Loops. At the end of the session, we provided the animals with a yogurt drop. These handling sessions lasted at least 10 min.

#### Virtual reality arena

Head-fixed mice ran atop a foam ball^96^ in a virtual reality environment with screens on each side and in front of their heads (Fig. 3a). Mice were first acclimatized to the ball without visual stimuli (two daily sessions, 45 min), while water was delivered at random intervals. Mice were then trained to lick when one of two stimuli appeared in random order in the virtual reality corridor every 30–70 cm of running selected from a uniform distribution. Reward probability was 50%. During the first two to three sessions, rewards were delivered automatically when the mouse ran through both cues. In later sessions, water rewards were delivered only in response to answer licks at the cues. After each behavioral session, water was supplemented to a total of 0.5–1 ml.

### Reporting summary

Further information on research design is available in the Nature Portfolio Reporting Summary linked to this article.

## Online content

Any methods, additional references, Nature Portfolio reporting summaries, source data, extended data, supplementary information, acknowledgements, peer review information; details of author contributions and competing interests; and statements of data and code availability are available at 10.1038/s41593-025-01923-4.

## Supplementary information

Supplementary Information Supplementary Tables 1–3 and text

Reporting Summary

Supplementary Video 1 Imaging turnover of single synapses. Imaging single synapses in layer 1 of cortex and in CA3 subfield of the hippocampus using ExM and Airyscan imaging. Related to Fig. 2k.

Supplementary Video 2 Intracellular pool of GluA2–HT in cortex. In green is the extracellular pool labeled by JF_549i_-HTL. In magenta is the intracellular pool labeled with JFX_673_-HTL. In cyan is nuclei stained with DAPI. Related to Fig. 4d.

Supplementary Video 3 Carotid artery infusion. Carotid artery infusion of dye ligand at a slow rate (20 µl min^−1^) followed by at a faster rate (40 µl min^−1^). Related to Extended Data Fig. 5.

Supplementary Table 3 MS results comparing WT to PSD-95-HT mouse protein turnover in the cortex.

Supplementary Table 4 Mice used in the study.

## Source data

Source Data Extended Data Fig. 10 Unprocessed western blots.

## Data Availability

Both metadata and raw data are available through the Open Science Foundation project (https://osf.io/wprhu/) associated with this paper. The protocols are available as a collection on protocols.io^97^. MS data were deposited at Mass Spectrometry Interactive Virtual Environment (MassIVE) under the identifier MSV000096857 (ref. ^98^) and ProteomeXchange under the identifier PXD059839. UniProt mouse (downloaded on 29 July 2023) was used as the database^93^. Source data are provided with this paper.

## Code availability

The code used for the analysis is available on GitHub at https://github.com/boazmohar/Unbiased/.
