# Supplementary material for: DELTA: a method for brain-wide measurement of synaptic protein turnover reveals localized plasticity during learning
Source: Nat Neurosci. 2025 Mar 31;28(5):1089–98. doi: 10.1038/s41593-025-01923-4 (PMC12081306; doi:10.1038/s41593-025-01923-4)
Supplement: Supplementary file 1 — Supplementary Tables 1–3 and text [file 41593_2025_1923_MOESM1_ESM.pdf]

nature neuroscience

Supplementary information https://doi.org/10.1038/s41593-025-01923-4

DELTA: a method for brain-wide measurement of synaptic protein turnover reveals localized plasticity during learning

In the format provided by the authors and unedited

Supplementary Table 1 – Animals and filters used for dye clearance experiments

ID	Date	Animal#	JF Dye	Obj.	Light source	Window
1	2019-03-22	444570	525	16x	1.5A Mightex 525	ALM
2	2019-03-25	439476	525	4x	1.5A Mightex 525	ALM
3	2019-03-26	439476	525	4x	1.5A Mightex 525	ALM
4	2019-03-28	434848	525	4x	3A Mightex 525	V1
5	2019-05-14	434848	552	20x	100% 565 coolLED	V1
6	2019-07-16	452088	669	4x	X-Cite 120	ALM
7	2019-07-17	452088	552	4x	5A Mightex 525	ALM
8	2019-07-18	452088	669	4x	X-Cite 120	ALM
9	2019-07-19	454527	552	20x	5A Mightex 525	ALM
10	2019-07-20	454814	669	4x	X-Cite 120	ALM
11	2019-07-22	452932	525	20x	5A Mightex 525	ALM

Supplementary Table 2: Animals and dyes used for ex vivo screening

Batch	Animal number	DOB	Virus injection date	HT-Expression	Dye injection	In-vivo dye	DMSO/PF127/ Saline/Injection (µl)	Chase dye	Mount	Fraction *in vivo*
2	439359	8/14/18	11/26/18	GFP	1/3/19	JF669	20/20/200/200	JF585	5th	0.47
2	439360	8/14/18	11/26/18	GFP	1/3/19	JF669	20/20/400/200	JF585	5th	0.43
3	444182	10/16/18	1/3/19	GFP	2/5/19	None	virus only	None	1	hist.
3	444185	10/16/18	1/3/19	GFP	2/5/19	JF585	30/10/200/200	JF669	5th	0.04
3	444186	10/16/18	1/3/19	GFP	2/5/19	JF585	10/30/200/200	JF669	5th	0.17
5	447231	12/4/18	4/5/19	GFP	5/1/19	JF669	20/20/200/200	JF585	4th	0.64
5	447232	12/4/18	4/5/19	GFP	5/1/19	JF669 2x	20/20/200/200	JF585	4th	0.66
5	447233	12/4/18	4/5/19	GFP	5/1/19	JF669 2x	20/20/200/200	JF585	4th	0.61
5	447234	12/4/18	4/5/19	GFP	5/1/19	JF669	10/30/200/200	JF585	4th	0.82
5	447235	12/4/18	4/5/19	GFP	5/1/19	JF669	10/30/200/200	JF585	4th	0.43
6	445766	12/5/18	4/12/19	GFP	5/8/19	JF552 2x	20/20/200/200	JF669	4th	0.54
6	445767	12/5/18	4/12/19	GFP	5/8/19	JF552 2x	20/20/200/200	JF669	4th	0.58
6	445768	12/5/18	4/12/19	GFP	5/8/19	JF552	20/20/200/200	JF669	4th	0.68
6	447239	12/4/18	4/12/19	None	5/8/19	JF552	20/20/200/200	JF669	4th	hist.
6	447240	12/4/18	4/12/19	GFP	5/8/19	JF552 2x	20/20/200/200	JF669	4th	0.61

7	452036	2/19/19	4/19/19	GFP	5/18/19	JF541	20/20/200/200	JF669	24th	0.03
7	452037	2/19/19	4/19/19	GFP	5/18/19	JF559	20/20/200/200	JF669	24th	0.03
7	452038	2/19/19	4/19/19	GFP	5/18/19	JF533	20/20/200/200	JF669	24th	0.02
7	452039	2/19/19	4/19/19	GFP	5/18/19	JF552	20/20/200/200	JF669	4th	0.37
8	452031	2/19/19	4/26/19	GFP	5/22/19	JF669	20/20/200/200	JF585	4th	0.87
8	452032	2/19/19	4/26/19	GFP	5/22/19	JFX612	20/20/200/200	JF585	4th	0.31
8	452033	2/19/19	4/26/19	GFP	5/22/19	552	20/20/200/200	JF669	4th	0.58
8	452034	2/19/19	4/26/19	GFP	5/22/19	608	20/20/200/200	JF552	4th	0.29
8	452035	2/19/19	4/26/19	GFP	5/22/19	Jfx608	20/20/200/200	JF552	4th	0.1
10	451116	3/11/19	7/15/19	mKate2	8/12/19	525	20/20/200/200	JF669	4th	0.33
10	451119	3/11/19	7/15/19	mKate2	8/12/19	525	20/20/200/200	JF669	4th	0.04
11	458564	5/21/19	8/8/19	GFP	11/11/19	JF669	20/20/200/200	JF585	4th	0.83
11	458565	5/21/19	8/8/19	GFP	10/24/19	JF669	20/20/200/200	JF552	4th	0.63
11	458566	5/21/19	8/8/19	GFP	10/24/19	JF646Bio	20/20/200/200	JF552	4th	0.04
11	458567	5/21/19	8/8/19	GFP	11/11/19	JF552	20/20/200/200	JF669	4th	0.2
11	458568	5/21/19	8/8/19	GFP	10/24/19	JF570	20/20/200/200	JF669	4th	0.05
13	460147	7/13/19		MeCP2-F	1/15/20	JF669	40/0/60/100	JF585	24th	
13	460149	7/13/19		MeCP2-F	1/15/20	JF669	20/20/60/100	JF585	24th	
13	467293	6/1/19		MeCP2-M	1/15/20	JF669	40/0/60/100	JF585	24th	
13	467294	6/1/19		MeCP2-M	1/15/20	JF669	20/20/60/100	JF585	24th	

13	467295	6/1/19		MeCP2-M	1/15/20	JF669	40/0/60/100	JF585	24th	
13	467296	6/1/19		MeCP2-M	1/15/20	JF669	20/20/60/100	JF585	24th	
14	456996	6/1/19		MeCP2-F	2/10/20	JF669	20/20/60/100	JF585	24th	
14	456997	6/1/19		MeCP2-F	2/10/20	JF669	0/40/60/100	JF585	24th	
14	456998	6/1/19		MeCP2-F	2/10/20	JF669	10/30/60/100	JF585	24th	
14	456999	6/1/19		MeCP2-F	2/10/20	JF669	0/60/40/100	JF585	24th	
14	457000	6/1/19		MeCP2-F	2/10/20	JF669	0/80/20/100	JF585	24th	

Supplementary Table 3 – MS results comparing WT to PSD-95-HT mice protein turnover in the cortex

Column	Title	Notes
A	**ID**	Protein Identifier
B	**Entry**	Database Entry
C	**Uniprot review state**	Reviewed is a high quality, manually annotated protein sequence database
D	**Entry Name**	Entry includes species
E	**Protein names**	Full protein name
F	**Gene Names**	Major gene name
G	**Length**	Protein sequence length (AA)
H	**WT 7 days**	H/L values for WT mice after 7 pulsing days
I	**WT 14 days**	H/L values for WT mice after 14 pulsing days
J	**PSD95-HaloTag 7 days**	H/L values for KI mice after 7 pulsing days
K	**PSD95-HaloTag 14 days**	H/L values for KI mice after 14 pulsing days
L	**half_life (WT)**	Calculated half-life for WT
M	**tHalf_c1 (WT)**	Lower limit of confidence interval for WT half-life
N	**tHalf_c2 (WT)**	Upper limit of confidence interval for WT half-life
O	**half_life (KI)**	Calculated half-life for KI
P	**tHalf_c1 (KI)**	Lower bound of confidence interval for KI half-life
Q	**tHalf_c2 (KI)**	Upper bound of confidence interval for KI half-life

R	**lifetime-tau (WT)**	Calculated tau for WT
S	**tau_c1 (WT)**	Lower bound of confidence interval for WT tau
T	**tau_c2 (WT)**	Upper bound of confidence interval for the WT tau
U	**lifetime - tau (KI)**	Calculated tau for KI
V	**tau_c1 (KI)**	Lower bound of confidence interval for KI tau
X	**tau_c2 (KI)**	Upper bound of confidence interval for the KI tau
Y	**log2(fold increase)**	Logarithm to base two of the ratio of KI to WT
Z	**-log10(p-value)**	Logarithm to base ten of the p-value of the comparison
AA	**pvalue**	Original p-value
AB	**Count WT**	Number of WT measures
AC	**Count KI**	Number of KI measures
AD	**Sum**	Sum of counts for WT and KI
AE	**Gene Ontology (cellular component)**	Major Gene Ontology annotation for the protein

Supplementary text

Simulations of pulse-chase experiments

We used simulations to gain an understanding of the conditions that would enable DELTA to provide precise measurements of protein turnover. We investigated multiple sources of error, including dye-ligand pharmacokinetics, amount of dye-ligand injected, variability in dye-ligand clearance, pulse-chase interval (ΔT), and compared DELTA to a pulse-only paradigm[29]. While the dye-ligand amount and ΔT are experimentally controlled, a crucial unknown is the pharmacokinetics of the HTL dyes. For example, if the free HTL dye is not cleared quickly, newly synthesized HT-protein could be labeled by the pulse dye-ligand instead of the chase dye, thus biasing the measurement to longer lifetimes (gray region in **Fig. 1a**).

We first measured the rate of dye-ligand clearance in the brains of wild-type (WT) mice. HTL JF dyes were injected into the retro-orbital sinus (**Extended Data Figure 1a; Methods**)[77] and imaging was subsequently performed with a wide-field fluorescence microscope[76] through a cranial glass window[99,100], for up to 24 hours after injection (**Extended Data Fig. 1b** for example imaging session). Dye-ligand concentration decayed with a dominant fast component (14±2 min),

and a smaller amplitude slow component (197±37 min; **Extended Data Fig. 1c-d** and **Supplementary Table 1**; fast/slow amplitude ratio: 1.7±0.3; n = 12; corresponding to a geometric average dye-ligand lifetime of 82 min). We incorporated dye-ligand clearance kinetics into our model (**Extended Data Fig. 2a**). Dye-ligand in a cytosolic compartment could either bind to the free protein-HT, directly clear out (corresponding to the fast component), or partition into a second compartment (lipids; corresponding to the slow component). We measured dye-ligand clearance in 12 mice. Fitting model parameters to each experiment (**Extended Data Fig. 2b**) produced 12 model variants that differ in the three parameters that impact dye-ligand clearance kinetics (lipids compartment volume, fast clearance rate constant, and cytosol to lipids equilibrium constant).

Because each HT-protein exists at different concentrations *in vivo*, it is difficult to inject the precise amount of HTL dye to reach saturation without leaving free dye-ligand. Thus, should we aim to under-saturate or provide dye-ligand excess? To address this question, we varied the ratio of dye-ligand to protein-HT target and estimated the error in estimating protein turnover for a broad range of average protein lifetimes (**Fig. 1b**). The dependence of the error on protein lifetime has three regimes. First, under-saturated labeling (**Fig. 1b** - dye/protein ratio < 1) results in large errors, regardless of protein lifetime. Second, dye-ligand saturation (**Fig. 1b** - dye/protein ratio > 1) results in large errors for short protein lifetimes, mirroring the under-saturation regime. Third, dye-ligand saturation results in small errors for long protein lifetimes (>40h). The main factor contributing to this effect in the dye-ligand saturating regime is the time of dye-ligand clearance relative to the measurement time. As the dye-ligand clears within an hour (**Extended Data Fig. 1**), the relative contribution to the error increases for shorter lived proteins, which entail shorter pulse-chase intervals. Specifically, in the saturating regime (>1.2 dye/protein ratio), dye-ligand clearance kinetics would bias the lifetime estimate towards larger values, as slower dye-ligand

clearance results in labeling of proteins synthesized after the pulse injection (gray shaded region in **Fig. 1a**). This would increase *Pulse* values and decrease *Chase* values, thus inflating the lifetime estimation. In a mild saturation regime (1-1.2 dye/protein ratio) during dye-ligand injection, new protein is made for shorter-lived proteins, thus more dye-ligand is consumed during the dye-ligand clearing window, which reduces the error introduced by dye-ligand excess. These competing factors create a nonmonotonic relationship between the estimation error and the protein lifetime (**Extended Data Fig. 2e**). Thus, two of the necessary conditions for reliably measuring the protein lifetime using DELTA are saturation of the target protein with pulse dye-ligand injection while avoiding very short-lived proteins. This is not very limiting as most proteins in the brain have lifetimes much longer than the one hour dye-clearance time[1,10].

We next measured the effect of model variants (combination of the lipids compartment volume, fast dye-ligand clearance rate, and cytosol-to-lipids equilibrium constant) on the estimation of protein lifetime across a wide range of simulated lifetimes (**Extended Data Fig. 2c)**. These variants capture the uncontrolled variable corresponding to the variance in dye-ligand clearance that would lead to variance (not bias) in our measurements. Using a pulse-chase interval equal to the average protein lifetime ($\Delta T = \tau$) and a pulse dye-ligand amount in the mildly saturating regime (1.2 dye/protein ratio), we compared the estimated lifetime with the true one and found a good correspondence (**Extended Data Fig. 2d**; r^2 = 0.9996 n = 84, 12 variants × 6 lifetimes). We identified a nonmonotonic relationship between bias and protein lifetime (Mean of error as defined above; **Extended Data Fig. 2e** – left panel), as expected from the single-model variant simulation. Of note, while bias (a shift of the mean) would lead to an overestimation of the lifetime, it does not affect our ability to make comparisons between brain regions in the same animal or between animals. Dye-ligand injection variability (Standard deviation of Error;

Extended Data Fig. 2e – right) would affect our ability to compare the lifetime and has a nonmonotonic shape as well. However, in all cases these were small (less than 5%).

Comparing DELTA with other protein turnover methods

We first compared DELTA with MS. MS results vary between individual studies depending on the cellular fraction selected and other experimental conditions (*e.g.*, *in vitro vs. in vivo*; for a review see Alvarez-Castelao & Schuman[101]), exhibiting considerable variability for any single protein, making comparisons across animals difficult. In our own experiments we observed that the HT fusion reduces PSD stability by approximately 2 days (**Extended Data Fig. 9i**), explaining some of the differences between DELTA and previous MS measurements. Additional differences might be related to the challenges in accounting for the recycling of isotope-labeled amino acids in MS experiments. A key advantage of DELTA over MS is the lack of recycling. Due to the enzymatic activity of HT on the HTL, a HT-protein that is degraded would release a ligand that can't rebind to a different unlabeled protein. Furthermore, as the chase HTL dye is delivered during perfusion, no degradation of the *Chase* is expected.

Next, we looked at other fluorescent imaging methods, which allow for labeling with different fluorophores at specific time points by fusing a tag with a protein of interest. This approach offers better spatial resolution, but remains difficult to deploy in the brain due to issues with epitope tags and labeled antibodies[102,103] or the self-labeling SNAP-tag[104] system and cognate ligands that do not cross the blood-brain barrier[105,106]. Lastly, other expression methods such as in *utero* electroporation to overexpress HT[59] or local injection of viruses to knock-in HT[107] does not allow sufficient spatial sampling to compare brain-wide turnover rates across mice.

We also compared DELTA to single-dye (pulse-only) methods[29,105]. First, we estimated the effects of the pulse-chase interval (Δt) in DELTA on the lifetime estimation error. While the

protein decayed exponentially (**Extended Data Fig. 2f** – black line), error decreased faster as function of Δt (**Extended Data Fig. 2f** – blue line). This decay of error again supported the idea that the main bias in this measurement is the relative time of dye-ligand clearance from the measurement time. Here, only one animal is modeled, so no averaging is used to calculate turnover rates. In contrast, pulse-only methods require multiple animals to determine the initial vs. later protein content of any given brain region. We modeled pulse-only experiments by assuming the same lifetime (100 h), a perfect measurement of the *Pulse*, and that the only variability would be across animals. We assumed that different animals would have on average 1 AU HT-protein and varied the coefficient of variation (CV 10-80%; **Extended Data Fig. 2g** – y axis). The range was selected conservatively according to the values seen in mass spectroscopy (MS) for biological replicates[108,109]**.** Variability could be reduced by averaging across multiple animals. We calculated the error in estimating the lifetime as a function of the number of animals used (**Extended Data Fig. 2g – x axis;** 4-72). These were distributed over four time points (0, 10, 30, 100h) in which a single exponential decay was fitted after averaging across animals (average number of animals per time point of 1, 3, 6, 12 animals). The estimation error increased with increasing protein expression variability and decreased with averaging over animals, as expected. However, for most operating regimes of DELTA, there would be no error under these conditions, as the *Fraction Pulse* calculation normalizes for the total protein amount.

We also investigated another source of error, photon shot noise. In pulse-only methods, lower expressing regions would reach the noise floor faster than higher expressing regions as *Pulse* signal declines with time. We simulated a pulse-only vs. pulse-chase experiments where signal to background ratio (SBR) was used as a proxy for expression strength (**Extended Data Fig. 2h)**. In each simulation we assumed perfect dye-ligand delivery and no variability in protein expression.

A baseline of 100 photons was used as a noise floor (to avoid counting errors). A protein (τ=2-200h; **Extended Data Fig. 2h** – x axis) was simulated using a signal of 100-1000 photons at t=0 representing SBR of 1-10 (**Extended Data Fig. 2h** – y axis) and measurements were taken at t=3.5, 7, 14 h. Mean errors of protein lifetimes were computed using bootstrapping and assuming Poisson noise. As expected, higher SBR reduced errors regardless of protein lifetime or methodology. However, DELTA was better in all simulated conditions (**Extended Data Fig. 2h)**. This has the prediction that low expression of a protein might have a negative effect on the ability to measure its turnover. Indeed, in publicly available data from Bulovaite et al.[29] there is a correlation between protein expression at day 14 and measured protein lifetime (**Extended Data Fig. 9f**). In contrast, we don't see such a relationship with the sum of the *Pulse* and *Chase* in DELTA (**Extended Data Fig. 9g**).

In DELTA, the *Pulse* + *Chase* accurately measures protein levels, allowing detection of changes across experimental conditions. These would mean an imbalance of synthesis and degradation, which would also lead to errors in turnover estimation that assume their balance. DELTA is able to offer a correction by estimating this error (**Methods**; **Extended Data Fig. 2i**). However, additional measurements are required to fully characterize the time varying synthesis and degradation rates.

Screening for bioavailable JF dyes

We screened HTL JF dyes that would be able to saturate abundant proteins in the brains of mice by measuring bioavailability in the brain (**Extended Data Fig. 3**). Our target was green fluorescent protein tagged with HT (GFP-HT). AAV-PHP.eB expressing GFP-HT was introduced by retro-orbital injection[81], which led to sparse and brain-wide expression. Four weeks after viral transduction, HTL dyes (**Supplementary Table 2**) were injected retro-orbitally. After 12-18

hours, we perfused the brain with a spectrally orthogonal dye. Given that GFP has a lifetime of several days[110], *Fraction Pulse* is a measure of the dye's ability to saturate the protein-HT target in the live animal.

The brain was sectioned coronally and imaged with a confocal slide scanner. Example images from an injection show the target protein in green (**Extended Data Fig. 3b**, left panel), the *in vivo* delivered dye-ligand in red (JF_{669}-HTL; **Extended Data Fig. 3b**, middle panel) and perfusion dye-ligand (JF_{585}-HTL; **Extended Data Fig. 3b**, right panel) in orange. This procedure enabled segmentation and analysis of single cells (**Extended Data Fig. 3c**). The fluorescence values of individual cells were background corrected and converted to dye-ligand concentration using calibration curves (**Extended Data Fig. 4a**). These calibrations are needed to determine the total protein amount (*Pulse* + *Chase*) correctly as the illumination and detection sensitivities of the dyes vary.

We identified two dyes that saturated GFP-HT in the brain: JF_{669}-HTL and JF_{552}-HTL. These dyes were significantly better than the other dyes tested (**Extended Data Fig. 3j**). Variability in GFP-HT expression did not account for the variability in these dyes, as the number of detected cells or virus signals did not correlate with Fraction Pulse (**Extended Data Fig 4b**). We also expect that a higher Fraction Pulse value would correlate with a higher *Pulse*/GFP ratio. Here, a large difference would implicate issues with the perfusion and complete saturation of the GFP-HT target with the *Chase*. However, Fraction Pulse did correlate with the *Pulse*/GFP ratio, as expected if the perfusion dye-ligand saturates the remaining GFP-HT proteins. This was the case both for JF_{669}-HTL and JF_{552}-HTL (**Extended Data Fig. 4c**).

We next quantified the amount of variability across brain regions in the delivery of the dye-ligand under these under-saturation conditions. We examined individual cells in all coronal

sections imaged (examples in **Extended Data Fig. 3d,e**). These injections produced low variability along the AP axis (**Extended Data Fig. 3f**), as indicated by normalizing the average Fraction Pulse in the front of the brain and the absence of any trend toward the back of the brain (**Extended Data Fig. 3g**). Looking at individual coronal sections (**Extended Data Fig. 3h**), for our bioavailable dyes we see a low CV for each slice (**Extended Data Fig. 3i**). This indicates low variability in dye-ligand delivery across the brain for JF_{669}-HTL and JF_{552}-HTL which would facilitate identification of variability in protein lifetime across brain regions (**Extended Data Fig. 3j**).

To assess successful saturation in a challenging case (MeCP2 is a very abundant protein, making it challenging to saturate in vivo), we injected JF_{669}-HTL retro-orbitally (*Pulse*) to the MeCP2-HT animal. This was followed by perfusion with JF_{585}-HTL (*Chase*) after 1 h (**Extended Data Fig. 7a**). The lack of *Chase* indicated saturation with the pulse dye-ligand in almost all tissues, including most regions of the brain (**Extended Data Fig. 7b–h**). Some brain regions containing dense cell body layers were not saturated, however, including the hippocampal CA1 region (**Extended Data Fig. 7i**). Nuclei located farther from the vasculature exhibited lower labeling intensities, suggesting this is due to dye-ligand depletion in regions with densely clustered nuclei expressing large amounts[44] of a HT fusion protein. Nevertheless, we achieved MeCP2–HT saturation in most brain regions (**Extended Data Fig. 7j,k**).

To assess the consistency of our method with MeCP2-HT, we looked at pairwise correlations across animals for all commonly imaged brain regions. The average mean correlation across pairs was high compared to shuffled controls for three levels of region annotation representing different spatial scales based on the Allen Brain Reference common coordinate framework (CCFv3; **Extended Data Fig. 8a–c**)[53].

Finally, we did not see a spatial correlation between the brain vasculature[111] and turnover measurements using the PSD-95-HT animal. For example, in both the hippocampus and neocortex, most blood vessels are oriented dorsoventrally while the pattern of turnover is mediolateral in the hippocampus (CA1 *vs.* CA3) and dorsoventral in neocortex (Layer 1 to Layer 6; **Fig. 2e**). This approach provides a good test case for biases that could arise from inhomogeneous delivery of dye-ligands via the vasculature, but we found that PSD-95 signal is homogenous at this resolution. As with MeCP2, we looked at pairwise correlations across animals for all commonly imaged brain regions. PSD-95-HT turnover was highly consistent across mice as the average mean correlation across pairs was high (**Extended Data Fig. 9d**).

Formulation and injection of bioavailable JF dyes

Janelia Flour (JF) HaloTag ligand (HTL) dyes are a key reagent for DELTA. We characterized these dyes in terms of infusion kinetics, formulation, solubility, and the brain's reaction in terms of inflammation. We first wanted to understand whether the injection rate can affect the dye's ability to saturate proteins in the brain. However, due to the retro-orbital injection procedure, we were unable to capture the dye-ligand dynamics during injection as the mouse headbar physically interfered with the retroorbital injection while it was mounted for imaging. Additionally, it is difficult to precisely control the rate of delivery in retroorbital injections, as they are performed manually. To perform these types of experiments we needed a precise i.v. dye-ligand delivery method that would enable simultaneous brain imaging. We choose to use carotid artery perfusion to deliver JF_{669}-HTL at different rates during continuous imaging through a cranial window (**Extended Data Fig. 5a**). In these experiments, if dye-ligand clearance is linearly proportional to the dye-ligand injection rate, then doubling the rate of dye-ligand delivery should exactly double the rate of dye-ligand increase in the brain. This would tell us that the dynamics of dye-ligand

injection would not dramatically change the total amount of dye-ligand delivered to the brain. However, if the slope of dye-ligand accumulation in the brain increases more than twofold, it would indicate a saturation of the dye-ligand clearance mechanism. This would favor faster dye-ligand injections leading to higher peak concentrations and total dye-ligand delivery to the brain. In the first mouse (**Extended Data Fig. 5b**) we started perfusion at 20 µl/min and later increased to 40 µl/min **(Supplementary Movie 3**). Increasing the delivery rate resulted in a more than twofold increase in the slope of dye-ligand accumulation in the brain (1.9 to 6.3 AU/min). We repeated this experiment with another animal (**Extended Data Fig. 5c)** and obtained similar results **(Extended Data Fig. 5d**). We validated our intuition about the effects of a constant clearing rate using simulations. Using the same amount of dye-ligand (20 AU) and a constant clearance rate (1 AU/dt), we simulated different injection rates (2-10 AU/dt). We observed saturation further away from the injection center for faster injection rates (**Extended Data Fig. 5e**). These results favor an injection method that delivers the dye-ligand faster given a constrained amount of dye.

To understand our dye-ligand constraints, we checked the dye-ligand solubility. We validated that our dyes are soluble at our desired concentration and stable over time (1 mM; **Extended Data Fig. 6e**). We used the published formulation for injection[76], which consists of 20 µl DMSO, 20 µl Pluronic F127, and 60 µl PBS. If we are near the solubility limit, the amount of dye-ligand in solution could decrease over time. This was not the case for all dyes injected *in vivo* (**Extended Data Fig. 6e**). The only dye-ligand that did not reach our solubility goal was JF_{585}-HTL, so it was not used *in vivo*. It was used during perfusion at a much lower concentration (1 µM) well below the solubility limit (~250 µM).

We then wanted to know if the ratio of DMSO to Pluronic F127 (1:1 20 µl each) was optimal for dye-ligand availability. We tested both increasing and reducing the ratio, but the original ratio

was the best (**Extended Data Fig. 6f**). As we were injecting 40-80 µl (~2-4 g/kg) of DMSO, which is lower than the LD50 (>10 g/kg)[112] but not a small amount, we tried to replace DMSO with Captisol as cosolvent and evaluated both the solubility and the in vivo injections. We saw mixed effects of dyes and formulations by looking at solubility after a 3-day incubation with DMSO, Captisol and a combination of Pluronic F127 and Captisol (**Extended Data Fig. 6g**). We tested both JF_{669} and JFX_{673} using retro-orbital injections with different formulations. As expected, from the solubility data, JF_{669} in other formulations was less bioavailable (**Extended Data Fig. 6h** – left column). However, although JFX_{673} was soluble in all formulations, it was still less bioavailable in the brain using Captisol (**Extended Data Fig. 6h** – Right column).

Finally, we validated that there were no signs of brain inflammation by staining for GFAP and Iba1 markers that we validated with a cortically lesioned animal (**Extended Data Fig. 6i -** panel i) and a naïve animal (**Extended Data Fig. 6i -** panel ii). Both retro-orbital virus injections (**Extended Data Fig. 6i -** panel iii) and our chosen dye-ligand injection formulation (**Extended Data Fig. 6i -** panel iv) did not affect these measures. This indicates that there is no major breach of the brain blood barrier, as it is known to induce this type of inflammation[113].

References

99. Holtmaat, A. *et al.* Long-term, high-resolution imaging in the mouse neocortex through a chronic cranial window. *Nat. Protoc.* **4**, 1128–1144 (2009).

100. Trachtenberg, J. T. *et al.* Long-term in vivo imaging of experience-dependent synaptic plasticity in adult cortex. *Nature* **420**, 788–794 (2002).

101. Alvarez-Castelao, B. & Schuman, E. M. The Regulation of Synaptic Protein Turnover. *J. Biol. Chem.* **290**, 28623–28630 (2015).

102. Butko, M. T. *et al.* Fluorescent and photo-oxidizing TimeSTAMP tags track protein fates in light and electron microscopy. *Nat. Neurosci.* **15**, 1742–1751 (2012).

103. Lin, M. Z., Glenn, J. S. & Tsien, R. Y. A drug-controllable tag for visualizing newly synthesized proteins in cells and whole animals. *Proc. Natl. Acad. Sci.* **105**, 7744–7749 (2008).

104. Keppler, A. *et al.* A general method for the covalent labeling of fusion proteins with small molecules in vivo. *Nat. Biotechnol.* **21**, 86–89 (2003).

105. Bojkowska, K. *et al.* Measuring In Vivo Protein Half-Life. *Chem. Biol.* **18**, 805–815 (2011).

106. Hawkins, B. T. & Egleton, R. D. Fluorescence imaging of blood-brain barrier disruption. *J. Neurosci. Methods* **151**, 262–267 (2006).

107. Willems, J. *et al.* ORANGE: A CRISPR/Cas9-based genome editing toolbox for epitope tagging of endogenous proteins in neurons. *PLOS Biol.* **18**, e3000665 (2020).

108. Walther, D. M. & Mann, M. Accurate Quantification of More Than 4000 Mouse Tissue Proteins Reveals Minimal Proteome Changes During Aging *. *Mol. Cell. Proteomics* **10**, S1–S7 (2011).

109. Zhao, Y., Denner, L., Haidacher, S. J., LeJeune, W. S. & Tilton, R. G. Comprehensive analysis of the mouse renal cortex using two-dimensional HPLC – tandem mass spectrometry. *Proteome Sci.* **6**, 15 (2008).

110. Verkhusha, V. V. *et al.* High Stability of Discosoma DsRed As Compared to Aequorea EGFP. *Biochemistry* **42**, 7879–7884 (2003).

111. Zhang, X. *et al.* High-resolution mapping of brain vasculature and its impairment in the hippocampus of Alzheimer's disease mice. *Natl. Sci. Rev.* **6**, 1223–1238 (2019).

112. Caujolle, F., Caujolle, D., Bouyssou, H. & Calvet, M. M. Toxicity and pharmacological aptitudes of Dimethylsulfoxide. *Comptes Rendus Hebd. Seances Acad. Sci.* **258**, 2224–2226 (1964).

113. Krenzlin, H., Lorenz, V., Danckwardt, S., Kempski, O. & Alessandri, B. The Importance of Thrombin in Cerebral Injury and Disease. *Int. J. Mol. Sci.* **17**, 84 (2016).
