# Supplementary material for: DELTA: a method for brain-wide measurement of synaptic protein turnover reveals localized plasticity during learning
Source: Nat Neurosci. 2025 Mar 31;28(5):1089–98. doi: 10.1038/s41593-025-01923-4 (PMC12081306; doi:10.1038/s41593-025-01923-4)
Supplement: Supplementary file 2 — Reporting Summary [file 41593_2025_1923_MOESM2_ESM.pdf]

nature portfolio

Corresponding author(s): Boaz Mohar

Last updated by author(s): Jan 17, 2025

Reporting Summary

Nature Portfolio wishes to improve the reproducibility of the work that we publish. This form provides structure for consistency and transparency in reporting. For further information on Nature Portfolio policies, see our Editorial Policies and the Editorial Policy Checklist.

Statistics

For all statistical analyses, confirm that the following items are present in the figure legend, table legend, main text, or Methods section.

n/a	Confirmed	
☐	☒	The exact sample size (*n*) for each experimental group/condition, given as a discrete number and unit of measurement
☐	☒	A statement on whether measurements were taken from distinct samples or whether the same sample was measured repeatedly
☐	☒	The statistical test(s) used AND whether they are one- or two-sided<br>*Only common tests should be described solely by name; describe more complex techniques in the Methods section.*
☒	☐	A description of all covariates tested
☐	☒	A description of any assumptions or corrections, such as tests of normality and adjustment for multiple comparisons
☐	☒	A full description of the statistical parameters including central tendency (e.g. means) or other basic estimates (e.g. regression coefficient) AND variation (e.g. standard deviation) or associated estimates of uncertainty (e.g. confidence intervals)
☐	☒	For null hypothesis testing, the test statistic (e.g. *F*, *t*, *r*) with confidence intervals, effect sizes, degrees of freedom and *P* value noted<br>*Give P values as exact values whenever suitable.*
☒	☐	For Bayesian analysis, information on the choice of priors and Markov chain Monte Carlo settings
☒	☐	For hierarchical and complex designs, identification of the appropriate level for tests and full reporting of outcomes
☒	☐	Estimates of effect sizes (e.g. Cohen's *d*, Pearson's *r*), indicating how they were calculated

Our web collection on statistics for biologists contains articles on many of the points above.

Software and code

Policy information about availability of computer code

Data collection	We used Tissuegnostics TissueFAXS SL verison 7 and Zeiss Zen blue version 3.4.WaveSurfer 1.0.2; Clampfit 11.3 ; Licor Image Studio 5.2.5
Data analysis	We used both python 3.7 and Matlab 2023b code available in the following link: https://github.com/boazmohar/Unbiased; QuickNII v2.2; VisuAlign v0.8;Prism 10; IP2, V6.5.5; ProLuCID V1.4.2;DTASelect V2.1.10; Census, V2.54.2.

For manuscripts utilizing custom algorithms or software that are central to the research but not yet described in published literature, software must be made available to editors and reviewers. We strongly encourage code deposition in a community repository (e.g. GitHub). See the Nature Portfolio guidelines for submitting code & software for further information.

Data

Policy information about availability of data

All manuscripts must include a data availability statement. This statement should provide the following information, where applicable:

- Accession codes, unique identifiers, or web links for publicly available datasets
- A description of any restrictions on data availability
- For clinical datasets or third party data, please ensure that the statement adheres to our policy

Both metadata and raw data are available through the Open Science Foundation project associated with this paper https://osf.io/wprhu/. The protocols are available as a collection on protocols.io. https://www.protocols.io/view/brain-wide-delivery-of-janelia-fluor-halotag-ligan-c9mnz45e. MS data was deposited at

Mass Spectrometry Interactive Virtual Enviroment (MassIVE) under the identifier MSV00009685797 and ProteomeXchange under the identifier PXD059839.D. UniProt mouse (downloaded on 07-29-2023) was used as the database.

Research involving human participants, their data, or biological material

Policy information about studies with human participants or human data. See also policy information about sex, gender (identity/presentation), and sexual orientation and race, ethnicity and racism.

Reporting on sex and gender	N/A
Reporting on race, ethnicity, or other socially relevant groupings	N/A
Population characteristics	N/A
Recruitment	N/A
Ethics oversight	N/A

Note that full information on the approval of the study protocol must also be provided in the manuscript.

Field-specific reporting

Please select the one below that is the best fit for your research. If you are not sure, read the appropriate sections before making your selection.

☒ Life sciences ☐ Behavioural & social sciences ☐ Ecological, evolutionary & environmental sciences

For a reference copy of the document with all sections, see nature.com/documents/nr-reporting-summary-flat.pdf

Life sciences study design

All studies must disclose on these points even when the disclosure is negative.

Sample size	No sample size calculation was done. We had the same number of animals as other protein turnover methods (see REF #11,29,85–87)
Data exclusions	No data was excluded.
Replication	Multiple animals were injected for each condition. For a detailed list see the source data with mouse identification. All attempts at replication were successful.
Randomization	Assignment was randomized.
Blinding	There was no blinding in this study. Blinding was not performed because the study relied on automated, quantitative measures (e.g., fluorescence intensity) that minimize observer bias.

Behavioural & social sciences study design

All studies must disclose on these points even when the disclosure is negative.

Study description	*Briefly describe the study type including whether data are quantitative, qualitative, or mixed-methods (e.g. qualitative cross-sectional, quantitative experimental, mixed-methods case study).*
Research sample	*State the research sample (e.g. Harvard university undergraduates, villagers in rural India) and provide relevant demographic information (e.g. age, sex) and indicate whether the sample is representative. Provide a rationale for the study sample chosen. For studies involving existing datasets, please describe the dataset and source.*
Sampling strategy	*Describe the sampling procedure (e.g. random, snowball, stratified, convenience). Describe the statistical methods that were used to predetermine sample size OR if no sample-size calculation was performed, describe how sample sizes were chosen and provide a rationale for why these sample sizes are sufficient. For qualitative data, please indicate whether data saturation was considered, and what criteria were used to decide that no further sampling was needed.*
Data collection	*Provide details about the data collection procedure, including the instruments or devices used to record the data (e.g. pen and paper, computer, eye tracker, video or audio equipment) whether anyone was present besides the participant(s) and the researcher, and whether the researcher was blind to experimental condition and/or the study hypothesis during data collection.*
Timing	*Indicate the start and stop dates of data collection. If there is a gap between collection periods, state the dates for each sample cohort.*

Data exclusions	*If no data were excluded from the analyses, state so OR if data were excluded, provide the exact number of exclusions and the rationale behind them, indicating whether exclusion criteria were pre-established.*
Non-participation	*State how many participants dropped out/declined participation and the reason(s) given OR provide response rate OR state that no participants dropped out/declined participation.*
Randomization	*If participants were not allocated into experimental groups, state so OR describe how participants were allocated to groups, and if allocation was not random, describe how covariates were controlled.*

Ecological, evolutionary & environmental sciences study design

All studies must disclose on these points even when the disclosure is negative.

Study description	*Briefly describe the study. For quantitative data include treatment factors and interactions, design structure (e.g. factorial, nested, hierarchical), nature and number of experimental units and replicates.*
Research sample	*Describe the research sample (e.g. a group of tagged Passer domesticus, all Stenocereus thurberi within Organ Pipe Cactus National Monument), and provide a rationale for the sample choice. When relevant, describe the organism taxa, source, sex, age range and any manipulations. State what population the sample is meant to represent when applicable. For studies involving existing datasets, describe the data and its source.*
Sampling strategy	*Note the sampling procedure. Describe the statistical methods that were used to predetermine sample size OR if no sample-size calculation was performed, describe how sample sizes were chosen and provide a rationale for why these sample sizes are sufficient.*
Data collection	*Describe the data collection procedure, including who recorded the data and how.*
Timing and spatial scale	*Indicate the start and stop dates of data collection, noting the frequency and periodicity of sampling and providing a rationale for these choices. If there is a gap between collection periods, state the dates for each sample cohort. Specify the spatial scale from which the data are taken*
Data exclusions	*If no data were excluded from the analyses, state so OR if data were excluded, describe the exclusions and the rationale behind them, indicating whether exclusion criteria were pre-established.*
Reproducibility	*Describe the measures taken to verify the reproducibility of experimental findings. For each experiment, note whether any attempts to repeat the experiment failed OR state that all attempts to repeat the experiment were successful.*
Randomization	*Describe how samples/organisms/participants were allocated into groups. If allocation was not random, describe how covariates were controlled. If this is not relevant to your study, explain why.*
Blinding	*Describe the extent of blinding used during data acquisition and analysis. If blinding was not possible, describe why OR explain why blinding was not relevant to your study.*

Did the study involve field work? ☐ Yes ☒ No

Reporting for specific materials, systems and methods

We require information from authors about some types of materials, experimental systems and methods used in many studies. Here, indicate whether each material, system or method listed is relevant to your study. If you are not sure if a list item applies to your research, read the appropriate section before selecting a response.

Materials & experimental systems

n/a	Involved in the study
☐	☒ Antibodies
☒	☐ Eukaryotic cell lines
☒	☐ Palaeontology and archaeology
☐	☒ Animals and other organisms
☒	☐ Clinical data
☒	☐ Dual use research of concern
☒	☐ Plants

Methods

n/a	Involved in the study
☒	☐ ChIP-seq
☒	☐ Flow cytometry
☒	☐ MRI-based neuroimaging

Antibodies

Antibodies used	Rabbit Anti-NeuN Millipore Cat# ABN78 RRID:AB_10807945 1:250 Manufacturer did positive control using mouse brain nuclear extract.

Anti-Iba1 antibody [clone: EPR16588] Abcam Cat# ab178846 RRID: RRID:AB_2636859 1:250 Manufacturer claims:
- Recombinant format for unrivaled batch-batch consistency: no need for same-lot requests
- Validated on the Leica BOND™ RX automated IHC staining platform for Rabbit IHC
- Antibody clone EPR16588 is the most widely used clone for Iba1 on the market and is cited in >660 publications
- Specificity and sensitivity confirmed in IHC with multi-tissue microarray (TMA) validation

Anti-SOX10 antibody [clone EPR4007-104] Abcam Cat# ab180862 RRID:AB_2721184 1:250 Manufacturer claims: Suitable for mIHC, IHC-P, IHC-Fr and reacts with Mouse, Human, Rat samples. Cited in 10 publications. We have tested this species and application combination and it works. It is covered by our product promise
Goat anti-Rabbit AF488 Molecular Probes Cat# A-11008 (RRID: AB_143165) 1:500 Manufacturer claims: Cross Adsorption against human IgG, human serum, mouse IgG, mouse serum and bovine serum.
anti-PSD95 antibody Clone K28/43 Millipore Cat# MABN68 RRID: AB_10807979 1:250 https://neuromab.ucdavis.edu/datasheet/K28_43.pdf
Goat anti-mouse CF633 secondary Biotium Cat# 20121-1 RRID: AB_10854245 1:500 Manufacturer claims: Highly-cross adsorbed for specific staining with minimal background
Anti-GluA2 antibody clone L21/32 Antibodies Incorporated Cat# 75-002 RRID: AB_2232661 1:300 https://neuromab.ucdavis.edu/datasheet/L21_32.pdf It is KO validated, detects human, mouse, and rat GluA2/GluR2 glutamate receptor, and is purified by Protein A chromatography. It is great for use in EM, IHC, ICC, IP, WB.
For WB: anti GluA2 pAb homemade JH6773 1:1000
For WB: anti PSD95 Addgene #184184 clone K28/74R 1:1000 It is KO validated, detects human, mouse, and rat PSD-95 MAGUK scaffold protein.

Validation

All marked as recommended for IF by the manufacturer. PSD95 and GluA2 was knock-out validated by NeuroMab https://neuromab.ucdavis.edu/datasheet/K28_43.pdf and https://neuromab.ucdavis.edu/datasheet/L21_32.pdf see list above for details

Animals and other research organisms

Policy information about studies involving animals; ARRIVE guidelines recommended for reporting animal research, and Sex and Gender in Research

Laboratory animals

C57Bl/6J RRID:IMSR_JAX:000664, both male and female. For MeCP2-HT and PSD95-HT knock-in mice of either sex were used. However, no comparisons were made between males and females for MeCP2-HT mice. GluA2-HaloTag mice were generated at Janelia and will be deposited at JAX. Age and sex of animals are in the supporting files of the manuscript.

Wild animals

No wild animals were used in the study

Reporting on sex

Either sex were used. However, no comparisons were made between males and females for MeCP2-HT mice.

Field-collected samples

No field collected samples were used in the study.

Ethics oversight

Janelia Institutional Animal Care and Use Committee.

Note that full information on the approval of the study protocol must also be provided in the manuscript.

Plants

Seed stocks

Report on the source of all seed stocks or other plant material used. If applicable, state the seed stock centre and catalogue number. If plant specimens were collected from the field, describe the collection location, date and sampling procedures.

Novel plant genotypes

Describe the methods by which all novel plant genotypes were produced. This includes those generated by transgenic approaches, gene editing, chemical/radiation-based mutagenesis and hybridization. For transgenic lines, describe the transformation method, the number of independent lines analyzed and the generation upon which experiments were performed. For gene-edited lines, describe the editor used, the endogenous sequence targeted for editing, the targeting guide RNA sequence (if applicable) and how the editor was applied.

Authentication

Describe any authentication procedures for each seed stock used or novel genotype generated. Describe any experiments used to assess the effect of a mutation and, where applicable, how potential secondary effects (e.g. second site T-DNA insertions, mosiacism, off-target gene editing) were examined.
