# Supplementary figures and images for: DELTA: a method for brain-wide measurement of synaptic protein turnover reveals localized plasticity during learning
Source: Nat Neurosci. 2025 Mar 31;28(5):1089–98. doi: 10.1038/s41593-025-01923-4 (PMC12081306; doi:10.1038/s41593-025-01923-4)

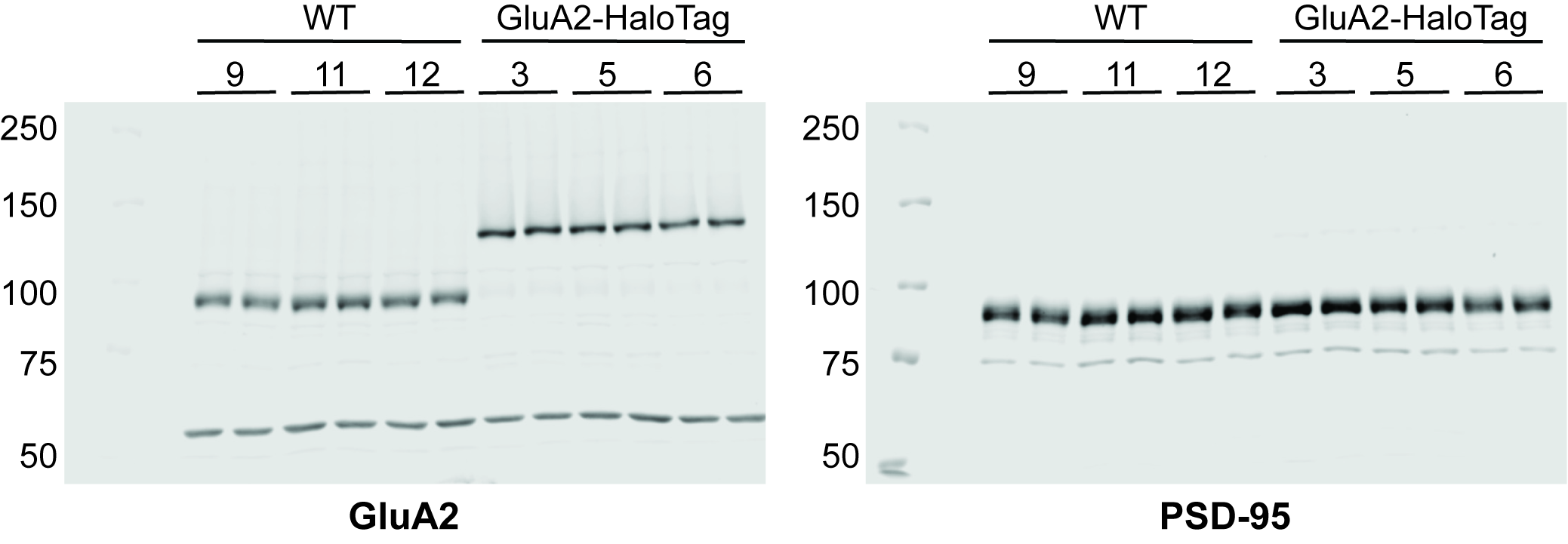

Supplement: Supplementary file 8 — Unprocessed western blots. [file 41593_2025_1923_MOESM8_ESM.tif]
